# Risky interpretations across the length scales: continuum vs. discrete models for soft tissue mechanobiology

**DOI:** 10.1007/s10237-021-01543-4

**Published:** 2022-01-05

**Authors:** Alberto Stracuzzi, Ben R. Britt, Edoardo Mazza, Alexander E. Ehret

**Affiliations:** 1 Empa, Swiss Federal Laboratories for Materials Science and Technology, Überlandstrasse 129, 8600 Dübendorf, Switzerland; 2 ETH Zurich, Institute for Mechanical Systems, Leonhardstrasse 21, 8092 Zürich, Switzerland

**Keywords:** Affinity, Discrete network, Continuum model, Collagen fibres, Scale-bridging, Soft biological tissues

## Abstract

Modelling and simulation in mechanobiology play an increasingly important role to unravel the complex mechanisms that allow resident cells to sense and respond to mechanical cues. Many of the in vivo mechanical loads occur on the tissue length scale, thus raising the essential question how the resulting macroscopic strains and stresses are transferred across the scales down to the cellular and subcellular levels. Since cells anchor to the collagen fibres within the extracellular matrix, the reliable representation of fibre deformation is a prerequisite for models that aim at linking tissue biomechanics and cell mechanobiology. In this paper, we consider the two-scale mechanical response of an affine structural model as an example of a continuum mechanical approach and compare it with the results of a discrete fibre network model. In particular, we shed light on the crucially different mechanical properties of the ‘fibres’ in these two approaches. While assessing the capability of the affine structural approach to capture the fibre kinematics in real tissues is beyond the scope of our study, our results clearly show that neither the macroscopic tissue response nor the microscopic fibre orientation statistics can clarify the question of affinity.

## Introduction

Soft biological tissues exhibit nonlinear anisotropic behaviour at the macroscopic ($\sim \text{mm}$) length scale analysed in ex vivo mechanical tests (e.g. Fung 1967). These distinctive characteristics result from the composition and assembly of their extracellular matrix (ECM), whose macromolecules are continuously remodelled by resident cells and organised to serve the tissue specific functions. The ECM transmits forces and displacements from the tissue boundary to the cell membrane, where complexes of membrane proteins further translate them to the intracellular structures (Humphrey et al. 2014). Cells are thus able to sense mechanical cues through the ECM (Humphrey et al. 2014) and respond by activation and regulation of cellular processes.

The understanding of how deformations are transferred from the organ and tissue (‘macroscopic’) length scale to the cell and ECM (‘microscopic’) scale of a few micrometers is therefore fundamental for a variety of mechanobiological challenges, such as the understanding of mechanics-driven regenerative or degenerative processes (Filippo and Atala 2002; Mazza and Ehret 2015). Existing experimental analyses of the length scale dependent deformation patterns suggest that this transfer is generally not uniform, neither in native nor engineered tissues, and that it can be characterised by both ‘attenuation’ and ‘amplification’ in strain (Upton et al. 2008; Han et al. 2013; Mazza and Ehret 2015).

Fibre-forming collagens interconnected in networks are recognised as the main structural solid component of the ECM in most tissues (Fratzl 2008), both in terms of volume fraction and mechanical contribution. Moreover, with regard to the cell-ECM interface, the transmission of displacements and forces between structural proteins and cells through focal adhesions represents the mechanical corner stone of one of the most established mechanotransduction pathways (Alenghat and Ingber 2002; Ingber 2006; Humphrey et al. 2014). The understanding of how collagen fibres deform within the deforming tissue therefore represents one of the essential aspects to bridge the gap between tissue-scale biomechanics and cell-scale mechanobiology.

The structural role of collagen has long been accepted in modelling, and several continuum theories have been proposed and profitably implemented to describe the macromechanical behaviour of fibrous soft tissues (see e.g. Chagnon et al. 2015; Lanir 2016). More recently, discrete approaches have been employed in biomechanics, which describe the microstructure by means of a large but finite set of ‘fibres’, typically modelled as springs or structural elements like rods or beams, randomly disposed in space. They are connected to form a network and loaded at the boundary of the definition domain (Stylianopoulos and Barocas 2007; Picu 2011). The huge number of fibres easily reachable with the typical sizes of soft tissue samples prevents from modelling full scale, complex geometries. Hence, the typical domain usually consists of a cuboid representative volume element (RVE), homogeneously loaded at the faces. The ‘macroscopic’ homogenised stress tensor can be computed by volume averaging (Picu 2011), enabling a direct comparison with continuum models and stress-strain curves obtained from experiments on tissues, but, in addition, these discrete network models (DNMs) integrate the underlying mechanisms at the fibre scale responsible for the tissue-scale response (Chandran and Barocas 2005).

Such computational models are increasingly used to rationalise the cause-effect nexus between mechanical cues and the corresponding cell reaction (Mak et al. 2015), to model mechanosensitive processes like cell contraction, alignment and migration (Vernerey and Farsad 2011; Obbink-Huizer et al. 2014; Kim et al. 2018), mechanical homeostasis (Eichinger et al. 2021), or to understand and guide the development of effective tissue equivalents, scaffolds and grafts in tissue engineering (Stella et al. 2008; Zündel et al. 2019; Domaschke et al. 2020). In view of the key role of fibre deformation as a scale-bridging element, it seems evident that the definition of a ‘fibre’ in these models and the assumed relation between fibre kinematics and macroscopic strain is a decisive factor in determining the reliability of these approaches.

Exactly with respect to fibre kinematics the vast majority of the current continuum approaches are distinct from the discrete ones: the former consider the tissue at a continuum length scale, largely neglect the real microstructure and model ‘fibres’ as vectorial line elements that represent directions of mechanical reinforcement, and whose deformation is completely predetermined through a kinematic relation that couples their change in orientation and shape with the gradient ${\mathbf {F}}(\varvec{ {X} },t)=\text{ Grad }{\varvec{\chi }(\varvec{ {X} },t)}$ of the macroscopic deformation ${\varvec{\chi }(\varvec{ {X} },t)}$ or its history. This includes the affine case where the fibre vectors are considered as material line elements that are mapped linearly by the deformation gradient (see e.g. Lanir 2016). This deterministic coupling implies that, under homogeneous loading conditions, fibres with equal reference orientation are mapped onto fibres with equal orientation in the deformed state. Conversely, in DNMs, the heterogeneous microstructure of the tissue is explicitly represented, even if in an idealised form. The loads applied at the boundary of the domain are transferred among the fibre elements through their interaction points (crosslinks), and the resulting deformations obey to the balance of forces and moments at these nodes and are then driven by energy minimisation (Chandran and Barocas 2005; Picu 2011). Hence, even under homogeneous affine boundary conditions, the deformations of the single fibre elements are generally non-affine and their relation with the macroscopic deformation is generally not unique. Notably, both the orientation distribution and the mechanical properties of the matrix affect the fibre kinematics (Hatami-Marbini and Picu 2009; Lake et al. 2011; Zhang et al. 2013). The case where the interaction between matrix and fibres dominates over the network effect has motivated alternative continuum models, e.g. based on fibres modelled as cylindrical inclusions in a soft matrix, that also serve to account for non-affine fibre kinematics (Morin et al. 2018; Bianchi et al. 2020). Generally, the questions of how much load is transferred through matrix and the fibre connections, respectively, and whether fibre kinematics are affine or non-affine in soft collagenous tissues have not been answered conclusively, and the answers may be highly tissue-specific.

The image-based analysis of the fibre kinematics in tissues, tissue-equivalents or tissue-engineering scaffolds remains inconclusive on whether the affine hypothesis applies or not, since the observed fibre reorientation upon macroscopic deformation could be explained successfully both in terms of affine (Lee et al. 2015; Stella et al. 2008) and non-affine models (Sander et al. 2009; Bircher et al. 2017; Ehret et al. 2017). Moreover, our own previous work has shown that both types of kinematics provide models able to fit the same sets of data from macroscopic material characterisation and generally capture the macroscopic behaviour, including even unusual characteristics of network materials (e.g. Buerzle and Mazza 2013; Bircher et al. 2017; Ehret et al. 2017; Domaschke et al. 2019).

Evidences to explain these apparent contradictions can be found in a revelatory study by Chandran and Barocas (2005), who compared the fibre kinematics of an illustrative 2D DNM when either affine boundary conditions were applied only at the RVE boundary or all fibres were forced to deform affinely. While, as expected, the predicted stretches experienced by the fibres were notably different and the stresses were markedly lower in the non-affine case, the analysis pointed at only small differences in the prediction of the averaged reorientation of fibres, concomitant with a lack of correlation between fibre orientation and stretch in the non-affine model (cf. Figs. 6, 7, 9 and 11 in Chandran and Barocas 2005). This aspect will be revisited and enhanced later in the present study. However, we start from a different setting: we use an established continuum constitutive model and an anisotropic 3D DNM that share (i) the form of the fibre orientation distribution in the reference state, (ii) the form of the fibre and matrix constitutive laws, and (iii) the macroscopic mechanical behaviour at tissue length scale. On the basis of these ‘equal competitors’, we investigate the quality of micro-to-macro and macro-to-micro predictions by exchanging material parameters between the two approaches, we shed light on the implications of the affinity assumption in the macro-to-micro strain transfer, and we discuss the results with regard to the interpretation of experimental data.

## Strategy of the study

**Fig. 1:**
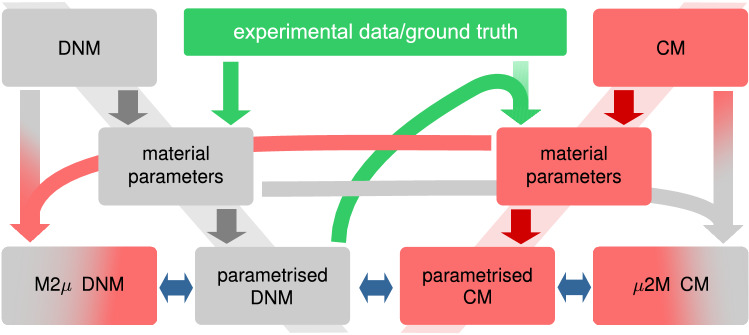
Block diagram illustration of the strategy adopted in the present study

The present study addresses (i) the capability of the CM and DNM approaches to capture the macroscopic response of soft biological tissue, (ii) the difference in their predictions to boundary value problems not used for parameter identification, (iii) the potential for predictions across the scales, and, finally, (iv) the fibre kinematics predicted by the two models. To this end, the following strategy is applied (Fig. 1). Based on the same constitutive law of matrix and fibres, and the same statistical functions describing the fibre orientation distribution, we define both a CM and a DNM. An existing data set on uniaxial (UA) monotonic extension on porcine pericardium (pPC) samples (Ehret et al. 2017) is used to calibrate the DNM, which then replaces the experimental data as new ground truth. The parametrised DNM is used to simulate equibiaxial extension (EB) and simple shear (SH). To address aspect (i), the CM is fitted and compared to the stress and lateral contraction responses in the UA and EB cases. To analyse the predictive qualities (ii), the SH response of the parametrised CM is compared to that of the DNM. The fitted parameters associated with the ‘fibres’ in the CM and DNM, respectively, are exchanged between the models for either micro-to-macro ($\mu$2M) predictions of the CM tissue response based on ‘true’ fibre properties, or macro-to-micro (M2$\mu$) predictions of the fibre and network scale characteristics implied by the fitted CM parameters (iii, iv).

## Common concepts

### Tissue-scale kinematics

Let the deformation of a material body ${\mathsf {B}}$ at time *t* be given through the mapping $\varvec{ {x} }=\varvec{\chi }(\varvec{ {X} },t)$ for all material particles of ${\mathsf {B}}$ with position vectors $\varvec{ {X} }$ and $\varvec{ {x} }$ in the reference and current configurations with respect to an arbitrary origin, respectively. The deformation gradient and its determinant $J=\det {\mathbf {{F}}}$ are defined by

1 $$\begin{aligned} \mathbf {{F}}(\varvec{ {X} },t) = \text{ Grad }{\varvec{\chi }}(\varvec{ {X} },t),\;\; J(\varvec{ {X} },t) = \det \mathbf {{F}}(\varvec{ {X} },t) \end{aligned}$$

and in what follows, the general dependence on position and time will be understood and the arguments omitted in writing. Finally, the right and left Cauchy-Green tensors are denoted by

2 $$\begin{aligned} \mathbf {{C}} = \mathbf {{F}}^{\mathrm T}\mathbf {{F}},\quad \mathbf {{b}} = \mathbf {{F}}\mathbf {{F}}^{\mathrm T}, \end{aligned}$$

respectively.

### Tissue free energy

Following the common modelling approaches, soft biological tissues are considered as composite materials of a non-fibrous matrix (m) and collagen fibres (f), disposed and interconnected to form a 3D network. When the tissue deforms, the fibres, here understood as the vectorial line elements between the crosslinks of the network, generally change in orientation and length. Let this length change be given by the ratio $\lambda$ of the fibre’s end-to-end length between the deformed and reference state, that will shortly be referred to as ‘fibre stretch’ in what follows. We consider the special case where the free energy $\varPsi _\mathrm{f}$ of a fibre per unit reference volume *V* of a collagenous tissue depends exclusively on the change of its end-to-end length, so that $\varPsi _\mathrm{f}$ can be expressed as a function

3 $$\begin{aligned} \varPsi _\mathrm{f} ={{\hat{\varPsi }}_\mathrm{f}(\lambda ) } = {\tilde{\varPsi }}_\mathrm{f}(\varLambda ), \quad {\hat{\varPsi }}_\mathrm{f},{\tilde{\varPsi }}_\mathrm{f}: (0,\infty ) \rightarrow [0,\infty ) . \end{aligned}$$

in terms of $\lambda$ or its square $\varLambda =\lambda ^2$. Such fibres can only transmit forces along the fibre axis, and thus form a central force network.

The overall free energy density $\varPsi$ of a tissue with such network microstructure consisting of *N* fibres results by summation of the individual contributions and addition of the matrix free energy density $\varPsi _\mathrm{m}$ per unit reference volume of the tissue *V*

4 $$\begin{aligned} \varPsi =\varPsi _\mathrm{m} + \varPsi _{\varSigma \mathrm f} =\varPsi _\mathrm{m}+\sum \limits _{k=1}^N \varPsi _\mathrm{f}^k\, , \end{aligned}$$

where the abbreviation $\varPsi _{\varSigma \mathrm f}$ has been introduced as the sum of the free energy densities of the *N* single fibres. For later use, we note that this cumulative fibre free energy density is equivalent to *N* times the arithmetic mean of the fibre free energy density

5 $$\begin{aligned} \varPsi _{\varSigma \mathrm f} =\sum \limits _{k=1}^N \varPsi _\mathrm{f}^k = N \sum \limits _{k=1}^N \frac{1}{N} \varPsi _\mathrm{f}^k = N \,\mathrm {E}[\varPsi _\mathrm{f}], \end{aligned}$$

and can thus be written as a multiple of the expected value $\mathrm{E}[\varPsi _\mathrm{f}]$.

### Fibre mechanical response

Let the free energy of a single fibre per unit reference volume *V* of the tissue be defined through a function $\varPsi _\mathrm{f}={\hat{\varPsi }}_\mathrm{f}(\lambda )$. Assuming that the former is determined through the product of a dimensionless, generally nonlinear function $G_\mathrm{f}={\hat{G}}_\mathrm{f}(\lambda )$ with properties

6 $$\begin{aligned} {\hat{G}}_\mathrm{f}(1) = 0,\quad \frac{\mathrm {d} {\hat{G}}_\mathrm{f}(\lambda )}{\mathrm {d} \lambda }(1) = 0,\quad \frac{\mathrm {d}^2{\hat{G}}_\mathrm{f}(\lambda )}{\mathrm {d}\lambda ^2}(1) = 4,\quad \end{aligned}$$

and a scalar factor $k_1'$ with dimension of stress, one may formally express the fibre free energy (3) as

7 $$\begin{aligned} \varPsi _\mathrm{f} = k_1'G_\mathrm{f}. \end{aligned}$$

Setting the non-dimensional stiffness (6)$_3$ equal to a positive constant makes the decomposition (7) unique.

The arbitrary choice ${\mathrm {d}}^2 G_\mathrm{f}/{\mathrm {d}} \lambda ^2(1)=4$ is merely a consequence of consistency with the particular constitutive model in Sect. 5.1.

The axial force acting on a fibre is then given by the fibre reference cross-sectional area ${A_\mathrm{f}}$ multiplied by the fibre nominal stress

8 $$\begin{aligned} f = {A_\mathrm{f}} \frac{V}{V_\mathrm{f}} \dfrac{\partial \varPsi _\mathrm{f}}{\partial \lambda } = \frac{V}{L_\mathrm{f}} k_1' \frac{{\mathrm {d}} G_\mathrm{f}}{{\mathrm {d}} \lambda } = K_1 \frac{{\mathrm {d}} G_\mathrm{f}}{{\mathrm {d}} \lambda } , \end{aligned}$$

where $V_\mathrm{f}$ and $L_\mathrm{f}$ denote the volume and length of a single fibre, and the factor $K_1={V}/{L_\mathrm{f}} k'_1$ with dimension of force has been introduced.

### Orientation distribution of fibres

Let the orientation of a fibre in the reference state be defined by a unit vector $\varvec{ {A} }\in {\mathcal {S}}$ on the three-dimensional unit sphere ${\mathcal {S}}=S^{2}$ that, with respect to an orthonormal basis $\{\varvec{ {e} }_x,\varvec{ {e} }_y,\varvec{ {e} }_z\}$ placed in the centre of the sphere, can be specified through the (modified) spherical coordinates $\phi \in [0,2 \pi )$, $\theta =\vartheta -\pi /2 \in [-\pi /2,\pi /2]$

9 $$\begin{aligned} \varvec{ {A} } =\cos \phi \cos \theta \varvec{ {e} }_x +\sin \phi \cos \theta \varvec{ {e} }_y +\sin \theta \varvec{ {e} }_z, \end{aligned}$$

where $\phi$ and $\vartheta$ denote the azimuth and polar angles, respectively. The orientation statistics of the large numbers of fibres ($N\gg 1$) is typically represented through a continuous orientation density function (e.g. Lanir 1979, 1983)

10 $$\begin{aligned} \varrho _{\varvec{ {A} }} = {\hat{\varrho }}_0(\varvec{ {A} }) = {\check{\varrho }}_0(\phi ,\theta ), \end{aligned}$$

such that the elemental probability ${\mathrm {d}} P_{\varvec{ {A} }}$ to find a fibre unit vector $\varvec{ {A} }$ within the elemental area ${\mathrm {d}} {\mathcal {S}} =\cos \theta {\mathrm {d}} \theta {\mathrm {d}} \phi$ of ${\mathcal {S}}$ is

11 $$\begin{aligned} {\mathrm {d}} P_{\varvec{ {A} }} = {\check{\varrho }}_0(\phi ,\theta ) \frac{1}{4 \pi } \cos \theta {\mathrm {d}} \theta {\mathrm {d}} \phi , \end{aligned}$$

and is subject to the normalisation condition

12 $$\begin{aligned} \int _{{\mathcal {S}}} {\mathrm {d}} P_{\varvec{ {A} }} = \frac{1}{4 \pi } \int _0^{2\pi } \int _{-\frac{\pi }{2}}^{\frac{\pi }{2}} {\check{\varrho }}_0(\phi ,\theta )\cos \theta {\mathrm {d}} \theta {\mathrm {d}} \phi = 1. \end{aligned}$$

When the tissue deforms, the fibres change their length and orientation, and their end-to-end vector changes from $\varvec{ {A} }$ to $\varvec{ {a} }'={\lambda }\varvec{ {a} }$, where $|\varvec{ {a} }|=1$. Hence, in the deformed current state at time *t*, the fibres are likewise characterised by an orientation distribution function. Upon expressing $\varvec{ {a} }$ with respect to the same spherical angles $\phi ,\theta$ in analogy to Eq. (9), the latter can be given by $\varrho _{\varvec{ {a} }}={\check{\varrho }}_t(\phi ,\theta )$. Importantly, however, the relations $\varvec{ {A} } \mapsto \varvec{ {a} }$, $\varvec{ {A} } \mapsto \lambda$ are neither deterministic nor one-to-one in a random network. Closed form relations between $\varrho _{\varvec{ {A} }}$ and $\varrho _{\varvec{ {a} }}$ therefore only exist for special cases such as the affine transformations considered in Rezakhaniha et al. (2012), Lee et al. (2015).

### Non-fibrous ECM

The Helmholtz free energy associated with the solid matrix is typically described phenomenologically in terms of isotropic hyperelastic constitutive models. Considering that biological tissues are highly swollen materials in vivo (Lanir 1987), and owing to the notable volume changes that the tissues can undergo during deformation by exchange of liquid with the environment (Ehret et al. 2017), here we used a chemomechanical isotropic compressible hyperelastic model, that represents the equilibrium case of a biphasic, osmotically active material (Stracuzzi et al. 2018). Its free energy is specified through a mechanical contribution $\varPsi _\mathrm{M}={\hat{\varPsi }}_\mathrm{M}(\mathbf {{C}})={\hat{\varPsi }}_\mathrm{M}(\mathbf {{b}})$ and a term ${\varPsi }_\mathrm{X}$ that accounts for the chemomechanical coupling (Hong et al. 2008; Ehlers et al. 2008; Ehret et al. 2017; Stracuzzi et al. 2018)

13 $$\begin{aligned} \varPsi _\mathrm{m} = {\hat{\varPsi }}_\mathrm{M}(\mathbf {{b}}) + \varPsi _\mathrm {X}(J), \end{aligned}$$

so that the Cauchy stress reads

14 $$\begin{aligned} \varvec{\sigma }_\mathrm{m} = \frac{2}{J} \frac{\partial \varPsi _\mathrm {m}}{\partial \mathbf {{b}}} \mathbf {{b}}=\frac{2}{J} \dfrac{\partial {\varPsi }_\mathrm{M}}{\partial \mathbf {{b}}}\mathbf {{b}} - p \mathbf {{I}}, \end{aligned}$$

where $\mathbf {{I}}$ denotes the identity tensor. In equilibrium, the chemical potential, whose gradient drives the fluid flow, vanishes, and the hydrostatic pressure *p* in Eq. (14) is balanced by the osmotic pressure difference $\varDelta \pi (J)$, so that $p=\varDelta \pi (J)$. The latter, in turn, calculates from $\varPsi _\mathrm{X}$ as (Stracuzzi et al. 2018)

15 $$\begin{aligned} \varDelta \pi (J) =-\frac{{\mathrm {d}} \varPsi _\mathrm {X}}{{\mathrm {d}} J} \end{aligned}$$

and satisfies the condition $\varDelta \pi (1)=0$.

## Continuum and discrete network models of fibrous tissues

Based on the common concepts outlined in Sect. 3, the modelling approaches can generally be differentiated into two main strategies: continuum mechanical and discrete computational approaches. Discrete models have gained increased interest in recent years, particularly to shed light on multi-scale deformation mechanisms and cell-scale events (Picu 2011; Beroz et al. 2017; Ehret et al. 2017; Zündel et al. 2019; Bircher et al. 2019; Eichinger et al. 2021). Continuum mechanical models, on the other hand, have successfully been used for decades, and among the various theories proposed, here we consider one variant of the affine structural model established in the 1970s (Lanir 1979, 1983), that has shown great versatility and whose offshoots have become a popular approach in soft tissue biomechanics (e.g. Sacks 2000; Li et al. 2018).

### Affine structural continuum model (CM)

Noteworthy, in the original approach by Lanir (1979, 1983) the presence of fibres with different lengths was accounted for by a first ‘averaging’ step, that collects the contribution of all fibres with different lengths but equal orientation (e.g. Eq. 34 in Lanir 1979). In view of Eq. (10), this implies that the vector $\varvec{ {A} }$ characterises the orientation of a whole family of fibres and that, accordingly, Eqs. (3) and (7) account for the lumped response of all fibres with equal orientation in the reference state.

#### Relation between macro- and micro-kinematics

A key question in continuum mechanical approaches is the relation between macroscopic (tisse-scale) and microscopic (fibre-scale) deformations, i.e. how orientation and length of a fibre change when the tissue is subject to a local deformation characterised by the deformation gradient $\mathbf {{F}}$. The affine structural model assumes that the unit vector $\varvec{ {A} }$ transforms like a material vectorial line element as

16 $$\begin{aligned} \mathbf {{F}}\varvec{ {A} } = \lambda _{\varvec{ {A} }} \varvec{ {a} }_{\varvec{ {A} }} \end{aligned}$$

with $\lambda _{\varvec{ {A} }}=|\mathbf {{F}}\varvec{ {A} }|$ and $|\varvec{ {a} }_{\varvec{ {A} }}|=1$. The subscript $(\cdot )_{\varvec{ {A} }}$ indicates that the stretch is completely determined by the deformation gradient and the referential direction $\varvec{ {A} }$ in this case. The squared stretch of a family of fibres initially aligned with the vector $\varvec{ {A} }$ is therefore given by the invariant

17 $$\begin{aligned} I_{\varvec{ {A} }} =\lambda _{\varvec{ {A} }}^2 = \mathbf {{C}}:\mathbf {{A}} \end{aligned}$$

of the two tensors $\mathbf {{C}}$ and $\mathbf {{A}}=\varvec{ {A} }\otimes \varvec{ {A} }$ (Spencer 1984).

#### Free energy and stress

Equating the fibre stretch by the affine stretch $\lambda _{\varvec{ {A} }}$ (16), and considering the result in (3), the free energy density of a fibre becomes a scalar invariant of $\mathbf {{C}}$ and $\mathbf {{A}}$, so that Eq. (3) reads

18 $$\begin{aligned} \varPsi _\mathrm{f} = {\tilde{\varPsi }}_\mathrm{f}(I_{\varvec{ {A} }}) = {\widehat{\varPsi }}_\mathrm{f}(\mathbf {{C}},\mathbf {{A}}). \end{aligned}$$

As a major consequence of the affine assumption, the fibre (family) stretch and thus its strain-energy density (18) for a given macroscopic deformation $\mathbf {{F}}$ are uniquely defined in terms of the orientation of $\varvec{ {A} }$. In view of the statistics of fibre stretch and energy, this implies that they are exclusively determined through the statistics of the referential orientation. The sampling space for a statistical sample thus becomes the unit sphere. In particular, the expected value of a fibre family’s free energy is obtained as an average over the fibre orientation distribution

19 $$\begin{aligned} \mathrm {E}[\varPsi _\mathrm{f}]= & {} \int \limits _{{\mathcal {S}}} \varPsi _\mathrm{f} \;\mathrm {d}P_{\varvec{ {A} }} \nonumber \\=& \frac{1}{4 \pi } \int _0^{2\pi }\int _{-\frac{\pi }{2}}^{\frac{\pi }{2}} {\hat{\varrho }}_0(\varvec{ {A} })\, {\widehat{\varPsi }}_\mathrm{f}(\mathbf {{C}},\mathbf {{A}}) \cos \theta {\mathrm {d}} \theta {\mathrm {d}} \phi , \end{aligned}$$

where we bear in mind that the components of $\varvec{ {A} }$ and $\mathbf {{A}}$ can be expressed in terms of $\phi$ and $\theta$. In accordance with Eq. (5), the cumulative free energy density provided by the CM thus takes a typical form of the strain-energy density associated with the fibres in the structural approach (Sacks 2000; Billiar and Sacks 2000; Chagnon et al. 2015)

20 $$\begin{aligned} \varPsi _{\varSigma \mathrm f}^\mathrm{CM}&= N \mathrm {E}[\varPsi _\mathrm{f}] \nonumber \\&= \frac{N}{4 \pi } \int _0^{2\pi } \!\!\int _{-\frac{\pi }{2}}^{\frac{\pi }{2}} {\hat{\varrho }}_0(\varvec{ {A} })\, {\widehat{\varPsi }}_\mathrm{f}(\mathbf {{C}},\mathbf {{A}}) \cos \theta {\mathrm {d}} \theta {\mathrm {d}} \phi \nonumber \\&= \frac{N}{4 \pi } \int _0^{2\pi } \!\!\int _{-\frac{\pi }{2}}^{\frac{\pi }{2}} {\hat{\varrho }}_0(\varvec{ {A} })\, k_1'{\hat{G}}_\mathrm{f}(\sqrt{I_{\varvec{ {A} }}}) \cos \theta {\mathrm {d}} \theta {\mathrm {d}} \phi = N k_1' \mathrm {E}[G_\mathrm{f}] \end{aligned}$$

that was reformulated by use of (7) in terms of ${G}_\mathrm{f}= {\hat{G}}_\mathrm{f}(\sqrt{I_{\varvec{ {A} }}})$. The second Piola-Kirchhoff stress contribution of the fibre families as follows:

21 $$\begin{aligned} \mathbf {{S}}_{\varSigma \mathrm f}^\mathrm{CM}&= 2 \dfrac{\partial \varPsi _{\varSigma \mathrm f}^\mathrm{CM}}{\partial \mathbf {{C}}} \nonumber \\&= \frac{N}{2 \pi } \int _0^{2\pi }\int _{-\frac{\pi }{2}}^{\frac{\pi }{2}} {\hat{\varrho }}_0(\varvec{ {A} })\, \dfrac{\partial {\tilde{\varPsi }}_\mathrm{f}(I_{\varvec{ {A} }})}{\partial I_{\varvec{ {A} }}} \varvec{ {A} }\otimes {\varvec{ {A} }} \cos \theta {\mathrm {d}} \theta {\mathrm {d}} \phi \nonumber \\&= \frac{N}{4 \pi } \int _0^{2\pi } \!\!\int _{-\frac{\pi }{2}}^{\frac{\pi }{2}} {\hat{\varrho }}_0(\varvec{ {A} })\, \frac{k'_1}{\sqrt{I_{\varvec{ {A} }}}} \left. \frac{{\mathrm {d}} {\hat{G}}_\mathrm{f}(\lambda )}{{\mathrm {d}} \lambda }\right| _{\lambda =\sqrt{I_{\varvec{ {A} }}} } \!\!\!\! \!\!\!\! \!\!\!\! \varvec{ {A} }\otimes \! {\varvec{ {A} }} \,\cos \theta \, {\mathrm {d}} \theta {\mathrm {d}} \phi \end{aligned}$$

and upon addition of the matrix part (14) the tissue stress provided by the continuum model in equilibrium ($p=\varDelta \pi$) is thus given by

22 $$\begin{aligned} \varvec{\sigma }^\mathrm{CM} = \varvec{\sigma }^\mathrm{CM}_{\varSigma \mathrm f} + \frac{2}{J} \dfrac{\partial {\varPsi }_\mathrm{M}}{\partial \mathbf {{b}}}\mathbf {{b}} - \varDelta \pi (J) \mathbf {{I}}, \end{aligned}$$

where $$\varvec{\sigma }_{\varSigma \mathrm f}^\mathrm{CM} =J^{-1}\mathbf {{F}}\mathbf {{S}}_{\varSigma \mathrm f}^\mathrm{CM}\mathbf {{F}}^{\mathrm T}$$.

#### Numerical approximation

Integration of equations (20) and (21) is known to be cumbersome, and in many relevant cases not achievable analytically, depending on the functional form of the integrand, i.e. the product of orientation distribution density and fibre free energy (Alastrué et al. 2009; Ehret et al. 2010). As a consequence, numerical integration methods have to be employed to obtain acceptable approximate solutions by cubature on the sphere (see e.g. Dai and Xu 2013), so that

23 $$\begin{aligned} \varPsi _{\varSigma \mathrm f}^\mathrm{CM} \approx N\sum \limits _{k=1}^{M} \omega _k\, {\hat{\varrho }}_0(\varvec{ {B} }_k) {\widehat{\varPsi }}_\mathrm{f}(\mathbf {{C}},\mathbf {{B}}_k) , \end{aligned}$$

and

24 $$\begin{aligned} \varvec{\sigma }_{\varSigma \mathrm f}^\mathrm{CM}\approx & {} NJ^{-1}\! \sum \limits _{k=1}^{M} \omega _k\, {\hat{\varrho }}_0(\varvec{ {\varvec{ {B} }_k} })\, \, \! \frac{k'_1}{\sqrt{I_{\varvec{ {B} }_k}}} \left. \frac{{\mathrm {d}} {\hat{G}}_\mathrm{f}(\lambda )}{{\mathrm {d}} \lambda }\right| _{\lambda =\sqrt{I_{\varvec{ {B} }_k}} } \!\!\!\! \!\!\!\! \!\!\!\! \mathbf {{F}}\varvec{ {B} }_k \otimes \! \mathbf {{F}}\varvec{ {B} }_k, \end{aligned}$$

where $\varvec{ {B} }_k$, $k=1,2,...,M$ define a set of integration points on the sphere, $\omega _k$ denote weights with the property $\sum _{k=1}^M \omega _k=1$ and $\mathbf {{B}}_k = \varvec{ {B} }_k \otimes \varvec{ {B} }_k$. Given the typical *J*-shaped fibre constitutive laws and their nonuniform distribution in collagenous tissues, the integrand may be highly nonlinear and even non-analytical, e.g. piecewise defined to exclude compressed fibres (Li et al. 2018), or characterised by a pole to include finite extensibility (Menzel and Waffenschmidt 2009). Therefore, the discretised models (23) are in general not exact, and moreover need a careful consideration of potential induced anisotropy inherited from the orientation of the *M* directions of the integration scheme (see the discussions in Verron 2015; Itskov 2016). Nevertheless, detaching from the idea of spherical integration, the discretised versions of continuum models with spatially distributed fibres become models on their own, and the set of directions formerly representing the integration scheme becomes part of the modelling assumption. Independent of whether seen as cubatures of the continuous integrals or as models on their own, the discrete versions have received great attention and application in soft tissue biomechanics during the last decade. Among the various cubature methods, we will restrict to spherical *t*-designs, designed to integrate exactly polynomials of order $\le t$ (Hardin and Sloane 1996). They are characterised by equal weights $\omega _k=1/M$, so that

25 $$\begin{aligned} \varPsi _{\varSigma \mathrm f}^\mathrm{CM} \approx \frac{N}{M}\sum \limits _{k=1}^{M} {\hat{\varrho }}_0(\varvec{ {B} }_k) {\widehat{\varPsi }}_\mathrm{f}(\mathbf {{C}},\mathbf {{B}}_k). \end{aligned}$$

Implicit but worth noting is the fact that, if *M* is large, the arithmetic mean of the *M* sampling directions in Eq. (25) provides an estimate of *N* times the expected value $\mathrm {E}[\varPsi _\mathrm{f}]$.

### A discrete network model (DNM)

Several approaches have been proposed to model the network of collagen fibres by a connected set of discrete springs or structural elements. In this section, we resume a particular DNM, adopted from our previous research, and tailored for the application to flat collagenous tissues and membranes (Ehret et al. 2017).

#### Generation of the RVE

At first, we define cuboid-shaped RVEs of dimensions $b_{\mathrm {RVE}} \times b_{\mathrm {RVE}} \times t_{\mathrm {RVE}}$, and seed a random set of points at a density $\varrho _{\mathrm {xl}}$ that define the referential positions of crosslinks between collagen fibres. These are interconnected by 1D straight lines with connectivity $z=4$ by means of a random weighted process in Matlab$^\circledR$ (The Mathworks, version 2018a), steered by a target distribution of fibre length and orientation that defines the associated weight as

26 $$\begin{aligned} \varrho _\mathrm {f0} (L,\phi ,\theta ) = \varrho _L(L){\check{\varrho }}_0(\phi ,\theta ), \end{aligned}$$

in terms of the orientation distribution density (10), and a given distribution density of referential fibre length between 0 and $L_\mathrm{cut}$

27 $$\begin{aligned} \varrho _L(L) = \frac{\gamma (L)}{L^2 C_\mathrm{cut} }, \quad 0< L < L_\mathrm {cut}. \end{aligned}$$

The latter is prescribed in terms of the $\gamma$-distribution

28 $$\begin{aligned} \gamma (L) = \frac{1}{\varGamma (a) h^a}L^{a-1}\mathrm {e}^{-\frac{L}{h}}, \end{aligned}$$

with shape parameter *a* and scale parameter *h*, and renormalised by

29 $$\begin{aligned} C_\mathrm{cut} = \int _0^{L_\mathrm {cut}} \gamma (L) \mathrm {d}L. \end{aligned}$$

With these choices, the following normalisation condition holds on the sphere

30 $$\begin{aligned} \frac{1}{4\pi } \int _0^{L_\mathrm {cut}} \int _0^{2\pi } \int _{-\frac{\pi }{2}}^{\frac{\pi }{2}} \varrho _\mathrm {f0}(L,\phi ,\theta ) \, L^2 \cos {\theta } \mathrm {d}\theta \mathrm {d}\phi \mathrm {d}L=1, \end{aligned}$$

and the two decoupled functions in (26) can be viewed as the contribution to the probability elements (Fisher et al. 1987) of fibre length and orientation, defined by $\rho _L(L)L^2\,\mathrm {d}L$, ${\check{\varrho }}_0(\phi ,\theta )\cos {\theta }\mathrm {d}\theta \mathrm {d}\phi$, respectively.

Notably, by this procedure one generates a network of straight connectors instead of curvy fibres. These straight links representing the end-to-end vectors of the fibre segments between the cross-links will be referred to as fibres in what follows. In analogy to the continuum model, the fibre’s elastic behaviour originating from intricate mechanisms of bending and stretching will be described through the nonlinearity of the strain-energy function $\varPsi _\mathrm{f}$ in terms of its end-to-end stretch, according to Eq. (7, 8).

**Fig. 2:**
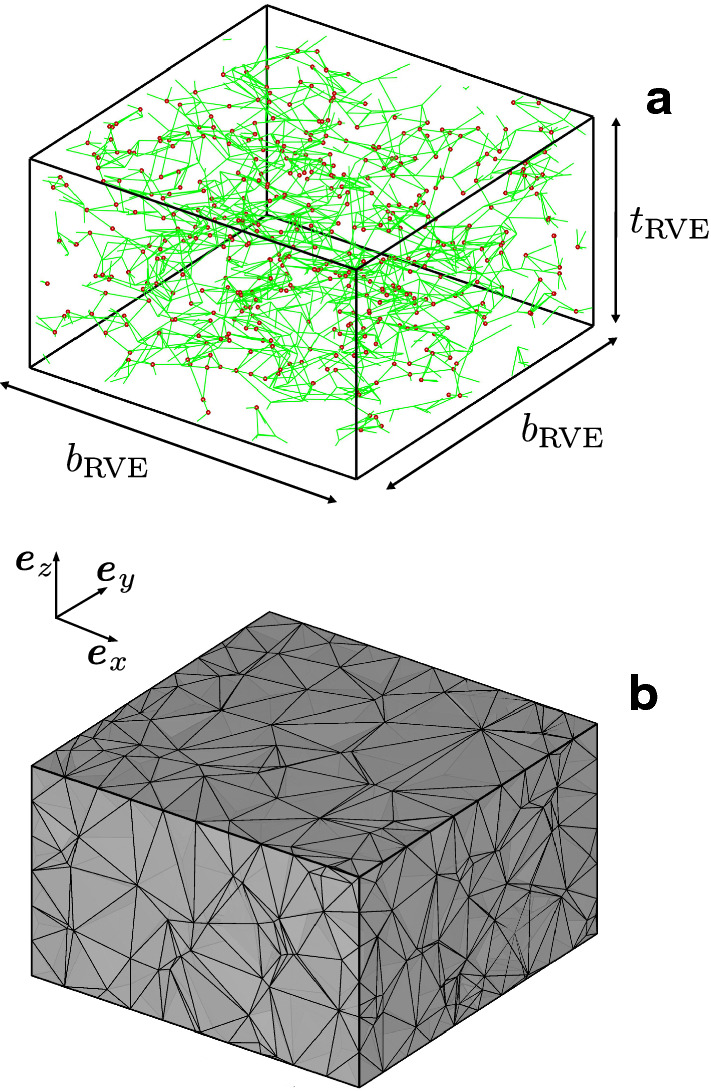
Example of discrete network architecture (**a**) and meshed RVE (**b**). The red spheres in (**a**) are plotted in the locations of the internal crosslinks used for the 3D triangulation procedure. For illustration purposes the images presented in this figure were generated with $b_{\mathrm {RVE}}/3$, $t_{\mathrm {RVE}}/3$, $L_\mathrm {fc}/3$, $\rho _{\mathrm {xl}} \times 2$ compared to the values specified in Sect. 4.2.1

The RVE is generated in Abaqus/Standard (Dassault Systèmes Simulia Corp, Providence, RI, USA) and each fibre is discretised by an axial connector element (CONN3D2). Finally, the mechanical contribution of the soft matrix is considered via tetrahedral solid continuum elements (C3D4) obtained by triangulating a subset of crosslinks of the RVE by means of a 3D Delaunay triangulation scheme. In particular, we considered 50% of the crosslinks (cf. Fig. 2) at which fibre connectors and continuum elements are tightly connected (Stracuzzi 2020). For the simulations presented herein the RVE parameters were specified as $b_{\mathrm {RVE}}= {250}{\mu }\mathrm{m}$ , $t_{\mathrm {RVE}}= {150}{\mu } \mathrm{m}$, $\rho _{\mathrm {xl}} = \mathrm {0.001275}\, \mathrm {nodes}/{\mu }\mathrm{m}^{3}$. Furthermore, the parameter of the length distribution density (28) were $a = 15$ and $h={1}{\mu } \mathrm{m}$, respectively, so that they define the mean connector length $L_\mathrm {fc} = ah = {15}{\mu } \mathrm{m}$, and $L_\mathrm {cut}=2 L_\mathrm {fc}$.

#### Fibre free energy and homogenised stress

The force-stretch law of the connector elements representing the fibres is prescribed by Eq. (8), so that for each fibre $i=1,2,...,N$ in the DNM the force is

31 $$\begin{aligned} f (\lambda _k) = K_1 \frac{{\mathrm {d}} {\hat{G}}_\mathrm {f}}{{\mathrm {d}} \lambda _k }. \end{aligned}$$

The elastic energy $w_\mathrm{f}^k$ stored in a single fibre that has changed its length from $L_k$ to $l_k=\lambda _k L_k$ reads

32 $$\begin{aligned} w_\mathrm{f}^k = \int \limits _{L_k}^{l_k} f(x) {\mathrm {d}} x = L_k \int \limits _{1}^{\lambda _k} f(y) {\mathrm {d}} y = L_k {{\hat{\psi }}}_\mathrm{f}(\lambda _k), \end{aligned}$$

where ${\hat{\psi }}_\mathrm{f}(\lambda _k)$ denotes the corresponding free energy per unit reference length of a fibre, and for the total network within the RVE domain consisting of *Q* fibres one obtains

33 $$\begin{aligned} w_\mathrm{\varSigma f}= & {} \sum _{k=1}^Q w_\mathrm{f}^k = \sum _{k=1}^Q L_k {{\hat{\psi }}}_\mathrm{f}(\lambda _k) \nonumber \\= & {} L_\mathrm{tot} \sum _{k=1}^Q \frac{L_k}{L_\mathrm{tot}} {{\hat{\psi }}}_\mathrm{f}(\lambda _k) = L_\mathrm{tot} \mathrm {E}[{{\hat{\psi }}}_\mathrm{f}], \end{aligned}$$

where ${L_\mathrm{tot}}=\sum _{k=1}^Q L_k$. Division by the volume $V_\mathrm{RVE}$ of the RVE provides the network’s free energy density contribution as introduced in (5)

34 $$\begin{aligned} \varPsi ^\mathrm {DNM}_{\varSigma \mathrm f} = \frac{w_\mathrm{\varSigma f} }{V_\mathrm{RVE}} = \frac{L_\mathrm{tot}}{V_\mathrm{RVE}} \mathrm {E}[{{\hat{\psi }}}_\mathrm{f}], \end{aligned}$$

and the tissue free energy density

35 $$\begin{aligned} \varPsi ^\mathrm {DNM} =\varPsi ^\mathrm {DNM}_\mathrm{m} + \varPsi ^\mathrm {DNM}_{\varSigma \mathrm f} \end{aligned}$$

is obtained after addition of the matrix contribution from the tetrahedral elements within the RVE.

The macroscopic deformations are imposed on the RVE by means of homogeneous boundary conditions, e.g. by prescribing the deformation of the RVE faces, and thus the displacements of the boundary nodes according to the corresponding deformation gradient $\mathbf {{F}}$. In the case of equilibrium at the crosslinks, i.e. zero internal forces, and quasi-static conditions, the RVE-averaged Cauchy stress is evaluated as (e.g. Chandran and Barocas 2005)

36 $$\begin{aligned} \varvec{\sigma } = \frac{1}{JV_\mathrm{RVE}}\sum \limits _{i=1}^{N_\mathrm{b}} \varvec{ {f} }_i\otimes \varvec{ {x} }_i, \end{aligned}$$

with $N_\mathrm{b}$, $\varvec{ {f} }_i$ and $\varvec{ {x} }_i$ the number of boundary crosslinks, their corresponding current force and position vectors, respectively.

### Matching DNM and CM in the affine case

The CM and DNM described in the previous sections share the mechanical behaviour of matrix and fibres as well as the orientation distribution of the latter discussed in Sects. 3.3–3.5. Nevertheless, given that the CM considers *N* fibres of unspecified length, whereas the RVE of the DNM contains *Q* fibres of various length between 0 and $L_\mathrm{cut}$ summing up to $L_\mathrm{tot}$, a reconciliating assumption is required to match the amount of fibre material present in both models, and thus to relate the constants $k_1'$ and $K_1$ in Eq. (8). To this end, we consider the hypothetical case where the fibres in the DNM undergo affine deformations, and match the free energy density with that of the affine CM. Imposing affine kinematics onto the crosslinks and thus the nodes of the tetrahedral elements of the RVE, the matrix free energy density $\varPsi _{\varSigma \mathrm m}$ is identical for the CM and DNM. As a consequence, our matching criterion simplifies to

37 $$\begin{aligned} \varPsi ^\mathrm {CM}_{\varSigma \mathrm f} \equiv \varPsi ^\mathrm {DNM}_{\varSigma \mathrm f}. \end{aligned}$$

Since each fibre connector with initial orientation $\varvec{ {A} }_k$ in the DNM is now mapped affinely, its length change is characterised by the stretch $\lambda _k=\sqrt{I_{\varvec{ {A} }_k}}$, and the energy per unit fibre length (32) thus reads

38 $$\begin{aligned} {\psi }^k_\mathrm{f} = \int _{1}^{\sqrt{I_{\varvec{ {A} }_k}}} f(\lambda ) {\mathrm {d}} \lambda = K_1 {\hat{G}}_\mathrm{f}(\sqrt{I_{\varvec{ {A} }_k}}). \end{aligned}$$

Substituting this result in (34), the linearity property of the expectation operator provides

39 $$\begin{aligned} \varPsi ^\mathrm {DNM}_{\varSigma \mathrm f} = \frac{L_\mathrm{tot}}{V_\mathrm{RVE}} \mathrm {E}[{{\hat{\psi }}}_\mathrm{f}] = \frac{L_\mathrm{tot}}{V_\mathrm{RVE}} \mathrm {E}[K_1 G_\mathrm{f}] = \frac{L_\mathrm{tot}}{V_\mathrm{RVE}} K_1 \mathrm {E}[ G_\mathrm{f}]. \end{aligned}$$

By direct comparison of (39) and (20) one finds

40 $$\begin{aligned} \frac{L_\mathrm{tot}}{V_\mathrm{RVE}} K_1 =Nk_1'=:k_1, \end{aligned}$$

where, for consistency with current approaches in soft tissue modelling, the number of fibres *N* was lumped into $k_1=Nk_1'$, whose units reflect the ‘energy stored in all fibres per unit volume of tissue’.

We emphasise that the imposition of affinity onto the fibre kinematics in the random network leads to additional forces at the crosslinks caused by the constrained kinematics. The affine case is thus generally not a minimiser of the (unconstrained) RVE’s free energy and it is therefore hypothetical.

### Distribution densities and image-based histograms of fibre orientation

Microscopy techniques and methods for image analysis are available to estimate the orientation of collagen fibres (Sander and Barocas 2009; Mauri et al. 2015), such as the ‘Directionality’ tool or the ‘OrientationJ’ plugin in ImageJ (Schneider et al. 2012; Rezakhaniha et al. 2012). The latter, for example, uses the local image structure tensor in a small domain, e.g. a single pixel, which contains information on the principal direction of orientation and the degree of alignment in the eigenvector corresponding to the maximal eigenvalue and the coherency, i.e. the ratio between difference and sum of the eigenvalues, respectively (Rezakhaniha et al. 2012). When applied over a larger region, e.g. the whole image, binning of the domain-wise outcomes with regard to the product of their orientation and coherency allows generating histograms of orientation ${\mathcal {R}}$ (Rezakhaniha et al. 2012), that provide an indication of the distribution of aligned structures within this region. Consequently the histograms represent the ‘fraction of fibre material per angle’. Literally, a longer fibre will generally occupy more pixels (or voxels in 3D) in an image than a shorter one of identical orientation. When using experimental histograms ${\mathcal {R}}(\phi ,\theta )$ to define the fibre distribution ${\check{\varrho }}_0(\phi ,\theta )$ in the reference state, this particularity is irrelevant for the models used in this study: since a longer fibre can be seen as a multiple of shorter fibres, this merely changes the meaning of the constant *N* in (5), which thus refers to the density of the ‘shorter fibres’. Moreover, in the proposed DNM the distributions of fibre length and orientation are independent of each other so that each direction is characterised by the same distribution of fibre length. However, the difference between the orientation distribution and the image histogram becomes consequential for the comparison between tissue data and models in the case where the fibre length correlates with the orientation. This situation concerns the deformed state of both DNM and CM, where the orientation distribution has changed from ${\check{\varrho }}_0(\phi ,\theta )$ to ${\check{\varrho }}_t(\phi ,\theta )$. While ${\check{\varrho }}_t(\phi ,\theta )$ reflects the realignment of the fibres, it does not account for the change of fibre material in a certain direction due to stretching. We therefore represent the distribution of orientation in a (current) length weighted form (cf. also Sander et al. 2009) so that

41 $$\begin{aligned} {\mathcal {R}}(\phi ,\theta ) \approx \ell (\phi , \theta ) {\check{\varrho }}_t(\phi ,\theta ). \end{aligned}$$

In the affine CM, we account of the change in fibre length through the affine stretch so that

42 $$\begin{aligned} \ell (\phi ,\theta ) = C_\mathrm{CM}^{-1}{\lambda }_{\varvec{ {A} }} \end{aligned}$$

where $C_\mathrm{CM}$ is a normalising constant. In the DNM we reconcile the two representations of orientation by weighting the orientation distribution ${\hat{\varrho }}_t(\phi ,\theta )$ predicted by the model by the fraction of fibre material aligned with a certain direction. Thus we have

43 $$\begin{aligned} \ell (\phi ,\theta ) = C_\mathrm{DNM}^{-1} \!\!\!\!\!\! \sum \limits_{\substack{k \\ \phi \le \alpha<\phi +{\mathrm {d}}\phi \\ \theta \le \beta <\theta +{\mathrm {d}}\theta}} \!\!\!\!\!\! l_k(\alpha ,\beta ) , \end{aligned}$$

where $C_\mathrm{DNM}$ is again a constant for normalisation and the sum adds the current lengths of all fibres $l_k(\alpha ,\beta )$ with orientation specified by the angles $\phi \le \alpha <\phi +{\mathrm {d}}\phi$ and $\theta \le \beta <\theta +{\mathrm {d}}\theta$. Since in the reference state fibre length and orientation are uncorrelated by (26), $l_k(\alpha ,\beta )$ in Eq. (43) becomes a constant, and ${\mathcal {R}}(\phi ,\theta )$ reduces to ${\check{\varrho }}_0(\phi ,\theta )$. To generate histograms for the affine model the 240 integration point set of a spherical 21-design (Hardin and Sloane 1996) was considered and subjected to an additional 100 random rotations to obtain 24240 sampling directions.

In-plane histograms ${\mathcal {R}}_{\phi }(\phi )$ were produced by mapping the 3D histograms ${\mathcal {R}}(\phi ,\theta )$ onto the plane $\theta =0$. The corresponding length weights therefore correspond to the projected lengths.

### Distribution of fibre stretch

Typically difficult to obtain in experiments but easily accessible in simulations, we also extracted the distributions of axial strain or stretch in the fibres (e.g. Chandran and Barocas 2005; Stylianopoulos and Barocas 2007; Sander et al. 2009; Mauri et al. 2016; Bircher et al. 2017). Slightly different from these fibre counts, here we use the length-weighted stretch distributions ${\mathcal {L}}(\lambda )$, that take into account the amount of material stretched. This concerns the DNM, where fibres have different length.

## A case study: porcine pericardium

**Fig. 3:**
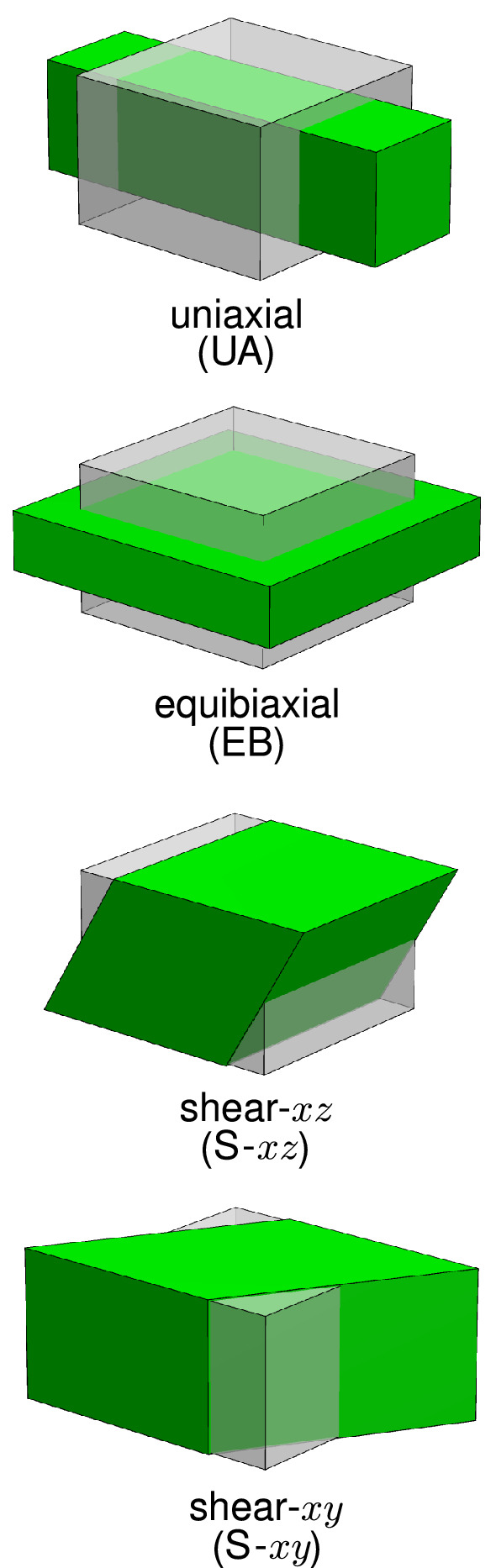
Sketches of the homogeneous deformation states used for numerical investigations: uniaxial (UA), equibiaxial (EB), simple shear in the *xz* (S-*xz*) and *xy* (S-*xy*) planes, respectively

With a thickness of 110–280${\mu }$m (Naimark et al. 1992; Gauvin et al. 2012; Caballero et al. 2017; Rassoli et al. 2019), pPC is a highly hydrated collagenous membrane enveloping the pig heart, with a water content of about 90% and a total collagen content per dry weight of around 70% (Naimark et al. 1992). Here we use an existing data set on pPC samples (Ehret et al. 2017) to specify the fibre and matrix characteristics and to identify the material parameters of the DNM and the CM. To investigate the role of the different fibre descriptions inherent to the CM and DNM with regard to the mechanical response, four homogeneous states of deformation (cf. Fig. 3) were simulated. Furthermore, EB extension of a membrane with a circular defect was studied as an example of a heterogeneous case (see Sect. 6.5).

### Model specification

**Fig. 4:**
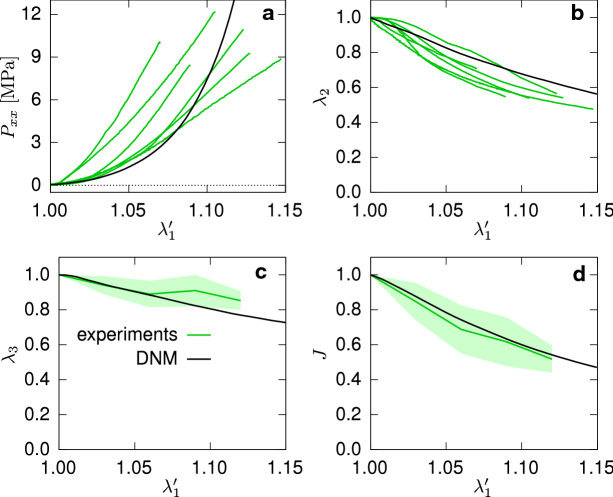
Nominal stress (**a**) and kinematics (**b**–**d**) in UA tests of pPC (experimental data from Ehret et al. 2017) and corresponding DNM (black solid lines). Data are represented with respect to a threshold force $F_{\mathrm {th}}=0.1 N$ with corresponding stretch $\lambda _\mathrm{th}$, and the abscissa was scaled accordingly so that $\lambda _1 = \lambda _1 ' \lambda _\mathrm{th}$. Green lines and shaded areas in (**c**,**d**) represent mean and ± standard deviation, respectively

In Ehret et al. (2017), pPC specimens were subjected to simple tension tests. The results contained in Fig. 4a demonstrate the typical, highly nonlinear *J*-shaped stress-strain response, characteristic of soft biological tissues, where the tension data in Ehret et al. (2017) was converted to stress for a typical thickness of 0.2mm.

Therefore we here describe the mechanical properties of the collagen fibres by the often used exponential strain-energy density function

44 $$\begin{aligned} \varPsi _\mathrm{f} = k_1'G_\mathrm{f} = \frac{k'_1}{2k_2}\left[ e^{k_2 \langle \lambda ^2-1 \rangle ^2}-1\right] \end{aligned}$$

according to Holzapfel et al. (2000), that specifies the CM. Macaulay brackets $\langle x\rangle = (x+|x|)/2$ are used to model negligible fibre contribution under compression. Eq. (44) implies the fibre force-stretch relation

45 $$\begin{aligned} f = K_1 \frac{{\mathrm {d}} G_\mathrm{f}}{{\mathrm {d}} \lambda } =2 K_1 \lambda \langle \lambda ^2-1 \rangle e^{k_2\langle \lambda ^2-1 \rangle ^2} \end{aligned}$$

that defines the fibre behaviour in the DNM.

The membranous structure of the tissue suggests to model the orientation distribution (10) by a decoupled representation

46 $$\begin{aligned} {\check{\varrho }}_0(\phi ,\theta )= \varrho ^0_\phi (\phi ) \varrho ^0_\theta (\theta ), \end{aligned}$$

such that $\phi$ specifies the angle within the membrane plane, and $\theta$ the out-of-plane angle, which is frequently applied to flat collagenous tissues (Holzapfel et al. 2015; Ehret et al. 2017). The comparison of bovine and porcine pericardia suggests that the anisotropy in pPC is less pronounced (Gauvin et al. 2012). Moreover, the data used herein (Ehret et al. 2017) do not specify the orientation with respect to the physiological directions of the tissue, so that conclusions on a potential in-plane anisotropy cannot be drawn. Therefore, and given that this point is not fundamental for the purpose of our study, in-plane isotropy is assumed, i.e.

47 $$\begin{aligned} \rho ^0_\phi (\phi ) = 1, \quad 0 \le \phi < 2\pi , \end{aligned}$$

while the out-of-plane distribution is specified in terms of a von-Mises-type distribution density, so that (cf. Holzapfel et al. 2015)

48 $$\begin{aligned} \rho ^0_\theta (\theta ) = 2 \sqrt{\frac{2b}{\pi }}\frac{\mathrm {e}^{\left\{ {b[\mathrm {cos}(2\theta )-1)]}\right\} }}{{\mathrm {erf}(\sqrt{2b})}}, \quad -\frac{\pi }{2} \le \theta < \frac{\pi }{2}. \end{aligned}$$

**Fig. 5:**
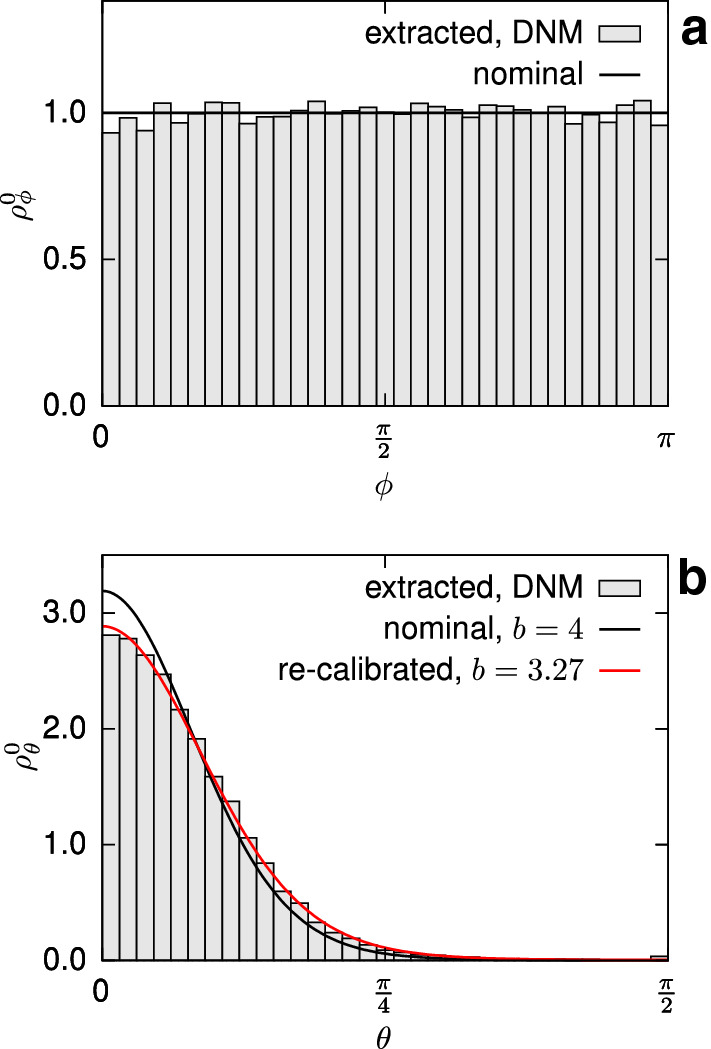
In-plane (**a**) and out-of-plane (**b**) fibre orientation density functions nominally imposed with the RVE generation algorithm (black curves) and extracted (histograms) from the actual RVE realisation. Histograms were mapped to the domain $0 \le \phi < \pi$, $0 \le \theta < \pi /2$, due to symmetry. The out-of-plane orientation function was re-calibrated (dashed red curve) to perform the simulations presented in Sect. 6.5.3

The functions (47) and (48) are plotted in Fig. 5a,b.

Similar to other collagenous membranes, pPC is characterised by strong lateral contraction and severe volume reduction under tensile load (Fig. 4b–d) and responds to changes of the osmolarity of the environment (Ehret et al. 2017). In line with these results, the matrix constitutive law (14) was therefore specified by (Ehret et al. 2017; Stracuzzi et al. 2018)

49 $$\begin{aligned} \varvec{\sigma }_\mathrm{m} = \frac{c}{J} \left[ \mathbf {{b}} -J^{2m} \mathbf {{I}} \right] - \varDelta \pi (J) \mathbf {{I}}, \end{aligned}$$

where

50 $$\begin{aligned} \varDelta \pi (J) = \pi (J) - \pi _0 = \pi _0\left[ \left( \frac{1-\phi _\mathrm {s}^\mathrm {ref} }{J-\phi _\mathrm {s}^\mathrm {ref}} \right) ^{\beta } - 1\right] , \end{aligned}$$

providing the osmotic pressure difference with respect to the reference value $\pi _0$ and with $\beta$ a nonlinearity parameter.

### Numerical implementations

All DNM simulations were run in Abaqus (Abaqus/Standard 6.14-1, Dassault Systèmes Simulia Corp, Providence, RI, USA) using the Python interface, and based on custom Fortran user material subroutine (UMAT) for the matrix contribution. For numerical stabilisation of the computations, a small additional damping contribution $d_\mathrm {c} \dot{\lambda _\mathrm {f}}$ ($d_\mathrm {c} = {0.001}{\mu }N s$) was added to the force (45) of each connector (cf. Mauri et al. 2016). The CM simulations in Sect. 6.5 were also simulated using Abaqus and adopting the same material subroutine for the matrix. The simulations of homogeneous deformation states of the CM as well as all the postprocessing analyses were performed with custom-made scripts using Matlab^®^ (version R2018a, The MathWorks, Inc.).

## Comparison of CM and DNM

### Calibration of the DNM

The DNM was calibrated against the UA tension data in terms of both nominal stress and kinematics (Fig. 4a,b,c) (Ehret et al. 2017). Since an approximate match of the response was sufficient for the scope of the study, the unknown parameters of the DNM were hand-tuned in order to represent the UA behaviour of pPC. In consideration of the observable experimental variability, acceptable agreement was obtained with the parameters listed in Table 1 in terms of the nonlinear stress-strain response and the reductions in width, thickness and, consequently, volume (black lines in Fig. 4).

**Fig. 6:**
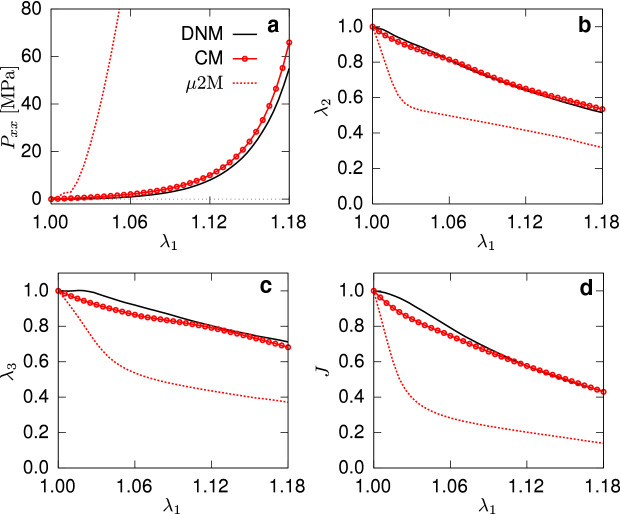
First Piola-Kirchhoff stress (**a**) and kinematics (**b**, **c**, **d**) in UA test for both the DNM (black) and CM (red)

**Fig. 7:**
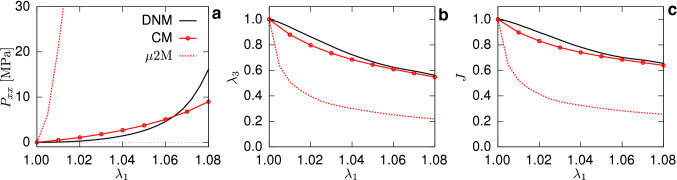
First Piola-Kirchhoff stress (**a**) and kinematics (**b**, **c**) under EB loading simulated with the DNM (black) and CM (red), respectively

The experimental data were given with respect to a small threshold load of 0.1N defining the reference state (Ehret et al. 2017), corresponding to the stretch $\lambda _1'=\lambda _1/\lambda _\mathrm{th}=1$ and a nominal stress threshold of 0.0333MPa for a typical thickness of 0.2mm. This threshold stress is also applied during the calibration of the DNM, which afterward provides access to the stress and kinematics responses starting from the zero-stress state, i.e. $\lambda _1=1$ (cf. black lines in Fig. 6). For this reason, and in view of the experimental scatter, the parametrised model was used as a new ground truth for reference, and in addition to the UA data, an EB data set was generated by a DNM simulation of the EB load case (Fig. 3) to serve as a basis for comparison with the CM (Fig. 7).

**Table 1:** Material parameters involved in the calibration of the models. Other parameters used in the simulations and equal for the two models: $\pi _0={0.001}\mathrm{MPa}$, $\beta =1.1$ , $\phi _\mathrm {s}^{\mathrm {ref}}=0.1$

Parameter	Units	DNM	CM
*c*	[MPa]	0.1	0.15803
*m*	[-]	1.8	2.2811
*b*	[-]	4$^*$	5.9865
$k_2$	[-]	19.3	21.4274
$K_1$	[N]	0.1	–
$k_1$	[MPa]	–	41.247

*Nominal value (cf. Fig. 5)

### Fitting of the CM

The CM was calibrated against the stress and kinematic responses of the parametrised DNM under UA and EB conditions by minimizing the objective function of the form

51 $$\begin{aligned} \sum _\kappa \sum _q e_q^\kappa , \quad e_q^\kappa = {w_q} \sum _{i=1} \left( \left. q_i^{\kappa } \right| _\text {{CM}} - \left. q_i^{\kappa } \right| _\text {{DNM}} \right) ^2 , \end{aligned}$$

where ${w_q}$ represent weight factors. Eq. (51) estimates the cumulative square error deriving from the point-to-point comparisons between the CM and the corresponding DNM curves for the *i*th $\lambda _1$, where $\kappa := \{\text{ UA,EB } \}$ identifies the type of deformation and $q:=\{\lambda _2, \lambda _3, P_{xx} \}$ the specific quantities considered, whose target curves are plotted in Figs. 6a–c and 7a,b. We note that, for the fitting procedure, no stress threshold was considered and also that in the EB load case $\lambda _2$ was prescribed and does not contribute to the error.

Noteworthy, not only the fibre-related parameters *b*, $k_1$ and $k_2$, but also *m* and *c* associated to the matrix had to be included in the optimisation procedure to obtain acceptable agreement with the DNM in both the UA (Fig. 6) and EB (Fig. 7) cases. Table 1 lists the parameters involved in the optimisation routine, while those common to the DNM and CM are given in the caption.

### Predicted macroscopic response in simple shear

To evaluate the predictive capabilities of the fitted CM in a different deformation state, we then simulated simple shear in the *xy* and *xz* planes with corresponding deformation gradients

52 $$\begin{aligned} \mathbf {{F}}_{\mathrm {S}\text {-}xy} = \mathbf {{I}}+\gamma \varvec{ {e} }_x\otimes \varvec{ {e} }_y \quad \mathbf {{F}}_{\mathrm {S}\text {-}xz} = \mathbf {{I}}+\gamma \varvec{ {e} }_x\otimes \varvec{ {e} }_z . \end{aligned}$$

**Fig. 8:**
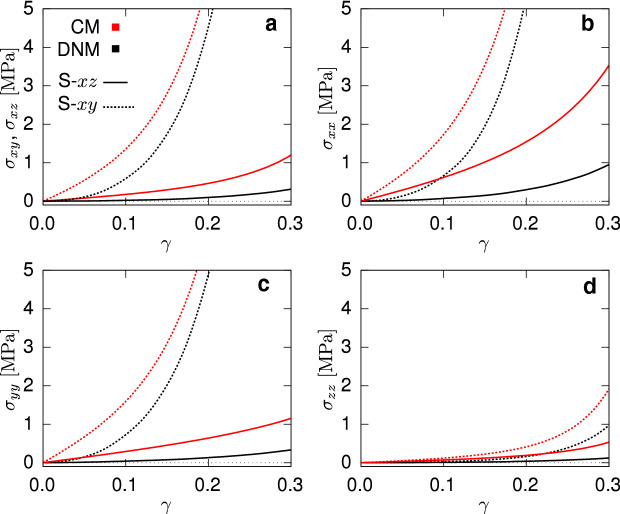
Shear (**a**) and diagonal (**b**, **c**, **d**) components of the Cauchy stress tensor in simple shear test for both the DNM (black) and CM (red)

For both the shear (Fig. 8a) and normal (Fig. 8b,c,d) components of the Cauchy stress, the results of the simulations, for both the DNM and CM, show an expected stiffer behaviour for the S-*xy* case compared to the S-*xz*, due to the pronounced in-plane orientation of the fibres (48). However, when comparing DNM and CM, considerable disagreement is observed for all the stress components. Drastic discrepancies are observed in the initial slope, i.e. the shear stiffness at small deformations, and in the nonlinearity of the curves. The results highlight that the ‘equivalence’ between the DNM and the CM, once matched with UA and EB data, does not hold in general at the macroscale for generic states of deformation.

### Micro-to-macro predictions

The fibre parameters of the calibrated DNM can be transferred to the CM to predict the tissue response based on ‘true’ fibre properties. To this end, the parameter $k_1$ of the CM was calculated through the relation (40), where the factor $L_{\mathrm {tot}}/ V_\mathrm {RVE}$ is obtained from the used RVE realisation of the DNM (see Table 1). Furthermore, given that in the DNM the RVE’s statistical realisation of the out-of-plane distribution is not perfectly matching the nominal one, a new value of the parameter *b* of the von-Mises function was determined from the extracted fibre distribution (see Fig. 5b) and adopted to provide a fair comparison. The simulations of UA (Fig. 6) and EB (Fig. 7) extensions show large differences between the DNM (black lines) and the $\mu 2 \mathrm {M}$ predictions (dashed red lines), for both stress and kinematics responses. Since the same microscale parameters were used to characterise the fibre distribution and mechanical response of the fibres, the observed mismatch provides a first indication of the impact of the affinity assumption intrinsic to the CM.

### Macro-to-micro predictions

Vice versa the calibrated CM provides another set of fibre-scale material parameters that define the constitutive behaviour of the fibres in this model. Moreover, the affine assumption predefines their reorientation with applied strain. These macro-to-micro (M2$\mu$) predictions were compared to the DNM results.

#### Predicted fibre behaviour

**Fig. 9:**
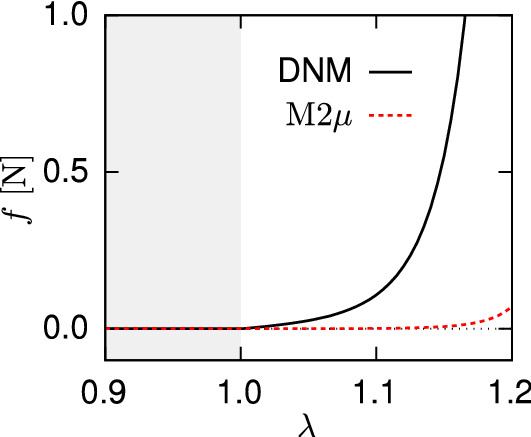
Force-stretch response of the fibre connectors in the DNM (black) and as obtained from the fitted CM (red)

Using Eq. (40), we calculated the single fibre stiffness parameter $K_1$ from the corresponding, fitted $k_1$ of the fitted CM, and plotted the corresponding force-stretch law of a single fibre (Fig. 9). The comparison with the analogous ground truth constitutive law implemented in the DNM reveals the considerable difference in the fibre elastic behaviour, with a M2$\mu$ prediction that is much softer than the ‘true’ one used in the calibrated DNM (cf. Fig. 9).

#### Predicted network kinematics in presence of a defect

**Fig. 10:**
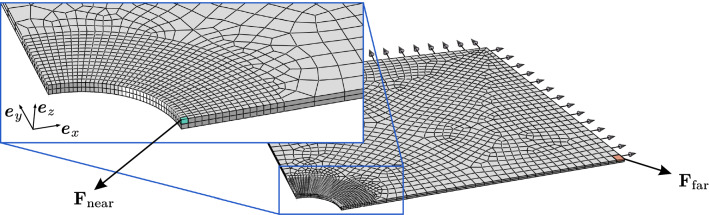
Computational model of a tissue sample with a circular defect. The deformation gradient tensor $\mathbf {{F}}$ is extracted in the near and far fields, and it is used to evaluate the distribution of orientation and stretch of the fibre connectors

We further compared the matched DNM and CM models in terms of the spatial orientation of the fibres in an inhomogeneous load state. We considered the EB extension of a tissue-scale square piece of membrane with a central circular defect of 1mm radius. Due to the symmetry of the material, load and geometry, we used and meshed only one octant of the full domain and applied symmetry boundary conditions (Fig. 10), resulting in a final side length and thickness of 10mm and 100${\mu }$m, respectively. The membrane was equibiaxially extended by 8$\%$ of its original dimensions and the local deformation gradient was extracted at the centroids of finite elements located at the defect (near field) and, for comparison, at the membrane boundary (far field), respectively. (cf. Fig. 10). With respect to the bases constructed from the vectors $\{\varvec{ {e} }_x,\varvec{ {e} }_y,\varvec{ {e} }_z\}$ of the global reference frame in Fig.10, the (rounded) components read

53 $$\begin{aligned}{}[{F}_{ij}]^{\mathrm {near}} = \begin{bmatrix} 0.810 &{} -0.007 &{} 0.005\\ -0.005 &{} 1.147 &{} 0.0\\ -0.018 &{} 0.0 &{} 0.580 \end{bmatrix}, \end{aligned}$$

54 $$\begin{aligned} \left[F_{ij}\right]^{\mathrm {far}} = \begin{bmatrix} 1.079 &{} 0.0 &{} 0.0\\ 0.0 &{} 1.080 &{} 0.0\\ 0.0 &{} 0.0 &{} 0.548 \end{bmatrix} . \end{aligned}$$

**Fig. 11:**
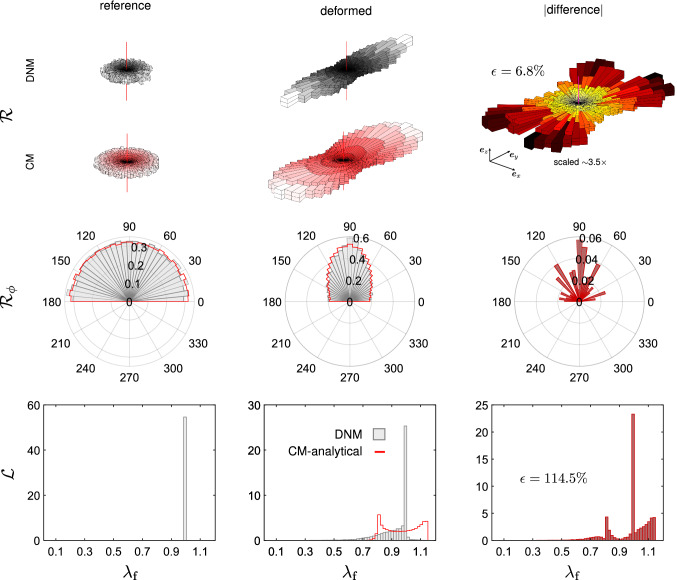
Fibre orientation histograms ${\mathcal {R}}$ (first row), ${\mathcal {R}}_\phi$ (second row) and stretch distributions (third row) in the reference (first column) and deformed (second column) configurations of an element extracted from the circular hole (cf. Fig. 10) and subjected to $\mathbf {{F}}_\mathrm {near}$. Data from DNM simualtions are reported as grey or grey-scale histograms. Data from the fitted CM are reported as red lines or red-scale histogram. In the last column, the corresponding absolute differences between DNM and CM data in the deformed configuration are reported

**Fig. 12:**
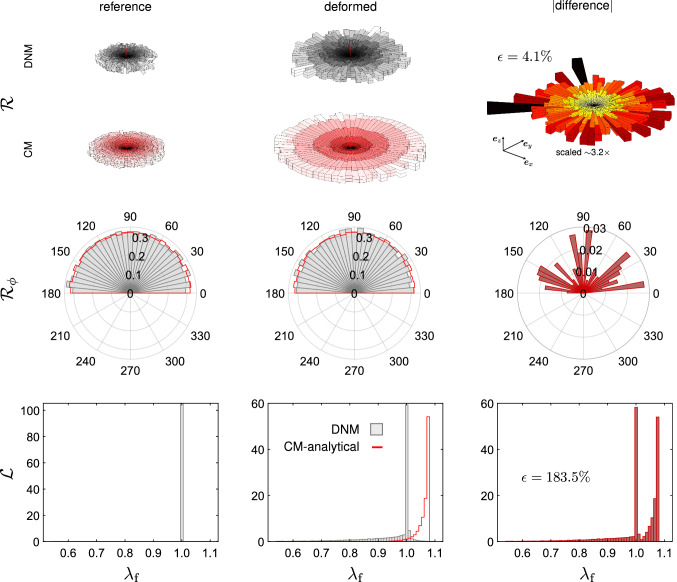
Fibre orientation histograms ${\mathcal {R}}$ (first row), ${\mathcal {R}}_\phi$ (second row) and stretch distributions (third row) in the reference (first column) and deformed (second column) configurations of an element extracted from the far field (cf. Fig. 10) and subjected to $\mathbf {{F}}_\mathrm {far}$. Data from DNM simualtions are reported as grey or grey-scale histograms. Data from the fitted CM are reported as red lines or red-scale histogram. In the last column, the corresponding absolute differences between DNM and CM data in the deformed configuration are reported

These two tensors were used to generate homogeneous boundary conditions for the DNM in two corresponding simulations. From these, we extracted the weighted distributions of the spatial ${\mathcal {R}}$ and in-plane ${\mathcal {R}}_\phi$ orientation, and we evaluated the distribution of the fibre stretch ${\mathcal {L}}$ (Figs. 11 and 12). For the sake of comparability, the 2D histograms are normalised such that $\int _I g(x) \mathrm {d}x = 1$, with *g*(*x*) the analysed distribution density of *x* and *I* its definition domain (‘pdf’ option in Matlab). The 3D spatial distributions ${\mathcal {R}}$ were evaluated in the domain $(\theta ,\phi ) \in [0,\pi /2] \times [0,\pi ]$ and then properly mirrored to get a 360$^{\circ }$ solid aspect.

The results for the near and the far field are reported in Figs. 11 and 12, respectively. The first column displays the distributions analysed in the reference configuration. The differently fitted parameters of the von-Mises distribution in the CM and DNM (cf. Table 1) lead to a slight mismatch of the out-of-plane distributions already in the reference state. In the second column, we show the orientation and stretch distributions extracted from the deformed configurations. The difference between the two models in terms of orientation is due to not only a propagation of the discrepancy at the reference, but also an effect of the different fibre motion. Most notable is the large difference between the models in terms of the stretch distribution (last row) ascribable to the affine vs. non-affine motions of the fibres in the two approaches. To better visualise the mismatch, we subtracted the histograms from each other and represented the absolute value of the difference in another histogram (last column). Adding up each bin’s volume (${\mathcal {R}}$) or area (${\mathcal {R}}_\phi$), respectively, and relating it to the corresponding values of the DNM provides a scalar measure $\epsilon$ to quantify the mismatch. Interestingly, despite the mismatch in the referential angular distribution between DNM and CM, the values of $\epsilon$ associated with the orientation distributions (6.8% and 4.1% for near and far field, respectively) are small, especially if compared to the corresponding values estimated for the stretch distributions (114.5% and 183.5%). This aspect is further analysed in the next paragraph, in which the source of error due to the fibre orientation in the reference configuration was properly eliminated.

#### Affine vs. non-affine DNM

**Fig. 13:**
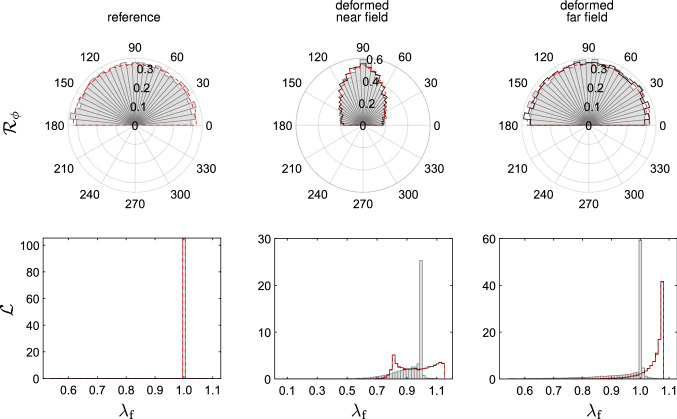
In-plane fibre orientation histograms ${\mathcal {R}}_\phi$ (first row) and stretch distributions (second row) in the reference (first column) and deformed configurations, near field (second column) and far field (third column). DNM simulations are reported as grey histograms, DNM simulations with affine deformations as black curves, and predictions of the CM with re-calibrated out-of-plane orientation ($b=3.27$, cf. Fig. 5b) as dashed red lines

In the previous analysis, the measured errors between DNM and CM in the fibre stretch and orientation distributions may not only result from the different deformation behaviour of the fibres, but also from the different parameters in the referential out-of-plane density functions between CM and DNM (cf. Table 1). To illustrate the isolated effect of the affinity assumption on kinematics, affine deformations were enforced in the DNM by prescribing the displacements of all crosslinks in the RVE in terms of the deformation gradients $\mathbf {{F}}_\mathrm {near}$ and $\mathbf {{F}}_\mathrm {far}$ (cf. Chandran and Barocas 2005). Interestingly, the in-plane orientation histograms in the deformed configurations are almost coincident for both the affine and non-affine DNMs (black solid lines and grey histograms, respectively, in Fig. 13). However, similar to Figs. 11 and 12, the corresponding fibre stretch distributions are considerably different between the two cases (last row in Fig. 13), thus revealing the distinctive motion of the fibres for each DNM.

For the sake of completeness, we also included the same results obtained with a CM for which we considered a parameter of the von-Mises distribution $b=3.27$, i.e. coincident with the best fit to the used DNM (see Fig. 5b). As expected, the results are essentially overlapping those obtained with the affine DNM (black vs. dashed red lines in Fig. 13).

## Discussion and conclusions

The DNM and CM presented in the previous sections are developed in a coherent way and share the same features. Specifically, the fibre orientation distributions describing the material anisotropy, the constitutive relations for matrix and fibres and the ‘fibre content’. However, the need for individual parameter identification to obtain the same response in homogeneous UA and EB states and the ample mismatch of the predictions in the shear states (cf. Fig. 8) evidence that the DNM and the CM do not describe the same material per se.

### Macro- and micro-behaviours are not bijective

The evident sound agreement between CM and DNM approaches when compared to a limited set of tissue scale experimental data (Figs. 6, 7) suggests that they have similar capacity in representing the macromechanical response of soft biological tissues. This is not surprising and in agreement with the vast amount of work that contributed to the advances in the biomechanics of soft biological tissues during the last decades. Given that, most probably, any CM would outperform a DNM in terms of computational efficiency, the effective CM approach would therefore remain the model of choice for many problems in tissue biomechanics.

However, the agreement in terms of the macroscopic response is in general not a sufficient condition to assume agreement in terms of micromechanics, i.e. the mechanisms of deformation at lower length scales. Vice-versa, furnishing the models with identical fibre-scale properties does not imply that the tissue-scale responses are in agreement. This is showcased by the $\mu$2M and M2$\mu$ predictions: the former demonstrates that, if the parameters of the DNM are transferred to an equivalent CM, the predicted stress responses for homogeneous deformations (cf. Figs. 6 and 7) are considerably higher than the DNM ones (cf. Chandran and Barocas 2005). Correspondingly, the M2$\mu$ analysis predicts a much softer response of a discrete ‘fibre’ (cf. Fig. 9), when the parameters of the calibrated CM are used to derive the force-strain law of an axial connector in the DNM. In view of the shared ingredients of the two models, this discrepancy must be attributed to the different fibre kinematics inherent to the two approaches.

### Affinity may not desrcibe the cell-scale environment

Continuum models with ‘fibres’ have been successfully used to rationalise ex vivo tests on soft biological tissues. At this length scale of analysis ($\sim \text{mm}$), the continuum hypothesis applies and the strain field is well approximated by affinity. Yet, microscopy studies reveal that the strain transfer across the scales in soft biological tissues is generally nonuniform (Upton et al. 2008; Han et al. 2013), so that deformations at the lower length scales can be highly heterogeneous and non-affine, and only become affine upon averaging over a critical length scale. This is consistent with the computational results of this study, as well as previous work (Mauri et al. 2016; Bircher et al. 2017; Chandran and Barocas 2005; Sander et al. 2009), that showed that the ECM deforms heterogeneously within the RVE and in particular that the fibres are subjected to stretches within a wide range, but of typically lower magnitude than the macroscopic principal stretches.

**Fig. 14:**
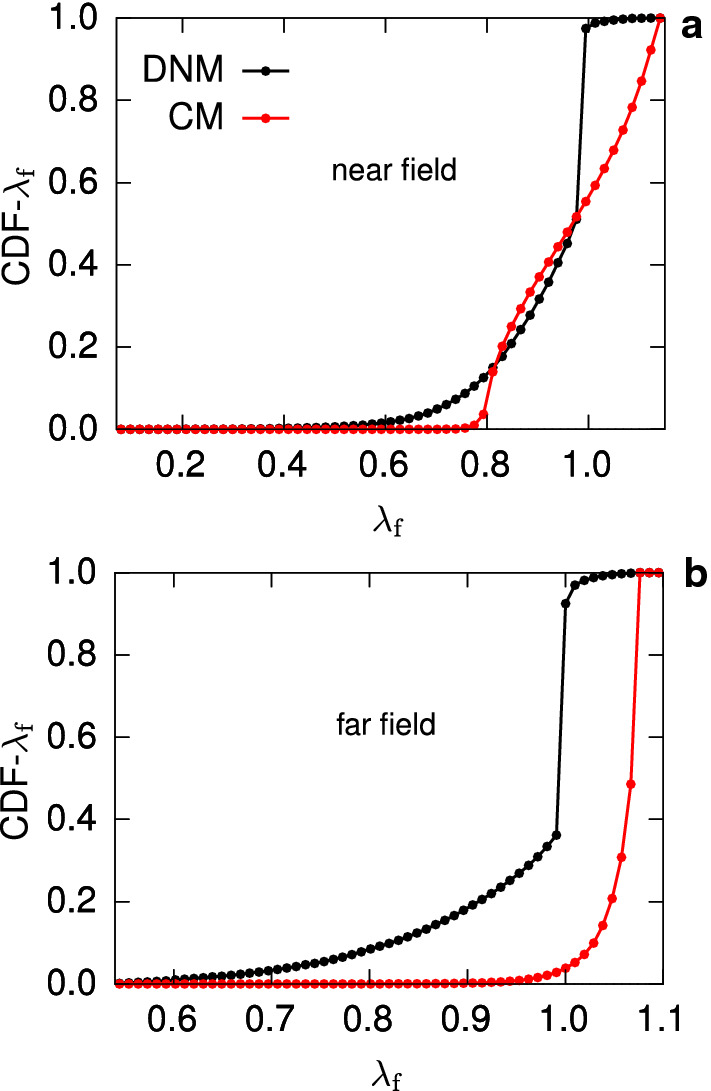
Cumulative distribution functions (CDFs) of the fibre stretch $\lambda _\mathrm {f}$ for the deformation states of the near field (**a**) and far field (**b**), deriving from the corresponding histograms plotted in Fig. 11 and Fig. 12. Black lines correspond to the DNM, red lines to the CM

Considered as an indicator of the error in predicting local strains, the stretch distributions obtained with our simulations (cf. second column, last row in Figs. 11 and 12) show a remarkable differences between the ‘true’ DNM (grey histograms) and the corresponding CM (red lines). In fact, in the DNM the fibre elements tend to accommodate the applied macroscopic deformation by reorienting and limiting their extension at the same time, thus explaining the peak of the stretch histograms around 1 (grey histograms in Figs. 11 and 12). More directly, the cumulative distribution functions (CDFs) derived from these histograms (Figs. 14a,b) demonstrate that the percentage of highly stretched ‘fibres’ is very small in the DNM, while it is much higher in the CM, even trespassing the 90% in the equibiaxially loaded far field (Fig. 14b).

**Fig. 15:**
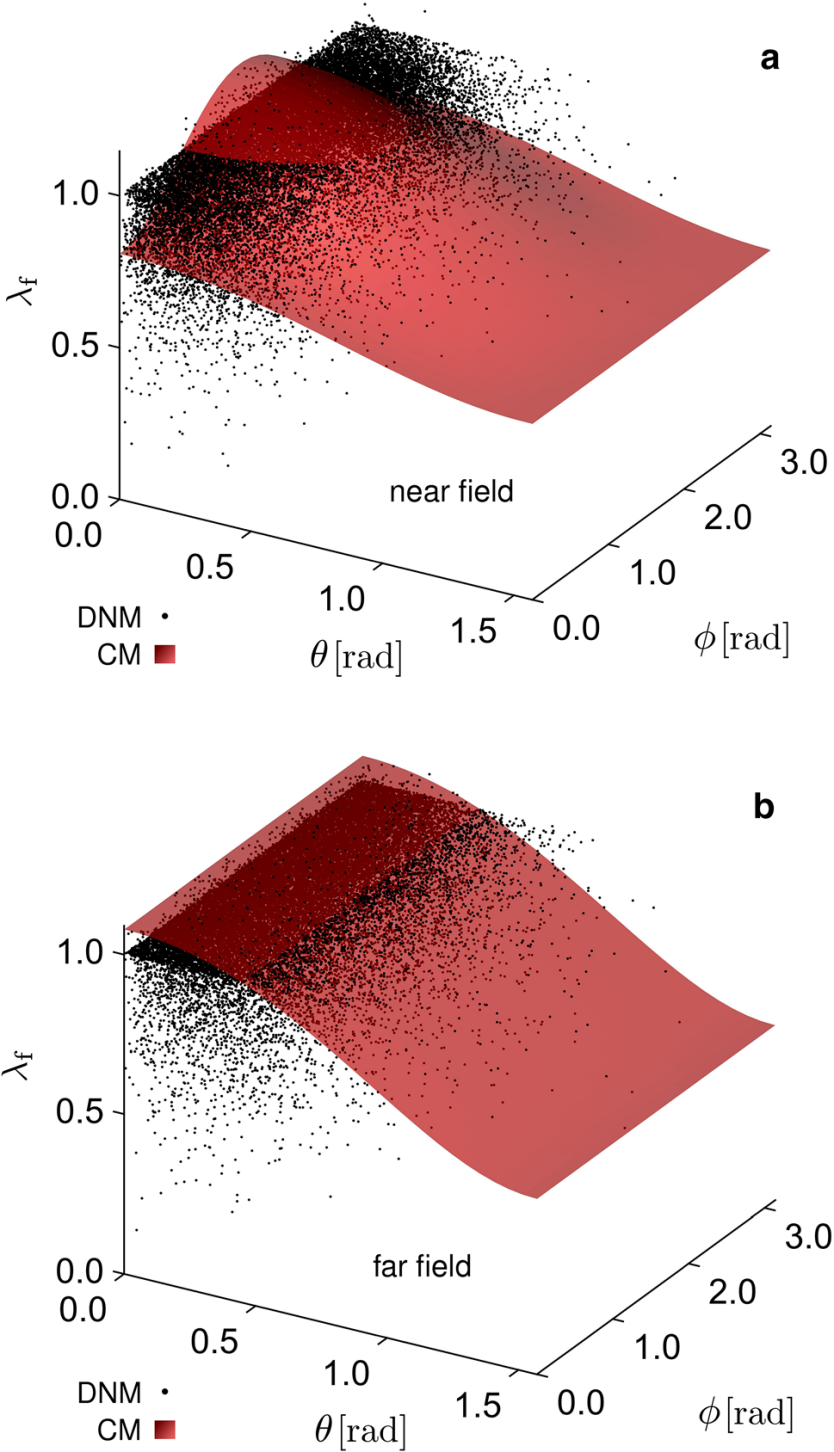
3D plots of the fibre stretch as a function of its reference spherical angles $\theta$ and $\phi$, for the DNM (clouds of black points) and the CM (smooth, shaded red surfaces), in the deformation states of the near field (**a**) and far field (**b**)

**Fig. 16:**
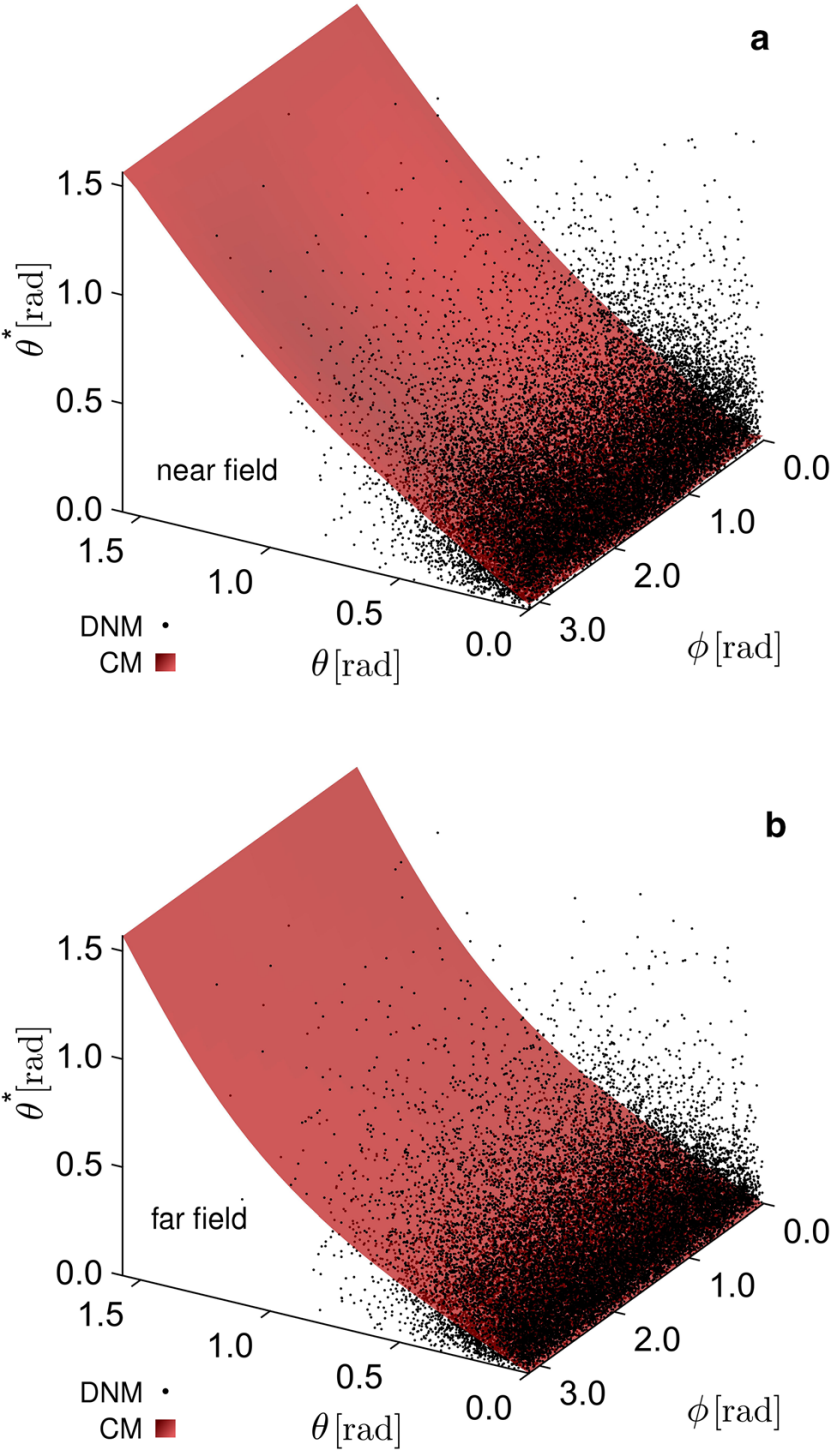
3D plots of the out-of-plane orientation $\theta ^*$ as a function of the reference spherical angles $\theta$ and $\phi$, for the DNM (clouds of black points) and the CM (smooth, shaded red surfaces), in the deformation states of the near field (**a**) and far field (**b**)

In addition to this, while in the CM the initial orientation is uniquely correlated with both the orientation and the stretch at a given deformation state, such a correlation is lacking in the DNM. In Figs. 15 and 16 we plot the stretch and the out-of-plane angle of each ‘fibre’ of the DNM, respectively, in the deformed states, near field (a), far field (b), as a function of the reference angles $\phi$ and $\theta$. For comparison, we also plot the predicted fibre stretch and out-of-plane angle in the deformed states, as prescribed from the affine assumption, i.e. $\lambda _\mathrm {f}(\theta ,\phi )=\sqrt{\mathbf {{C}}:\mathbf {{A}}(\theta ,\phi )}$ and $\theta ^*(\theta ,\phi )=\mathrm {arcsin}\left( \mathbf {{F}}\varvec{ {A} }(\theta ,\phi )\cdot \varvec{ {e} }_z / |\mathbf {{F}}\varvec{ {A} }(\theta ,\phi )|\right)$. The figures clearly indicate that, while the function $\lambda _\mathrm {f}(\theta ,\phi )$ and $\theta ^*(\theta ,\phi )$ describe smooth surfaces for the affine case, the corresponding values in the case of the DNM are highly scattered. This result is in line with the 2D analysis in Chandran and Barocas (2005, Figs. 8a, 9a therein).

The significant difference between the stretch distributions of continuum and discrete approaches, and the lack of a one-to-one correspondence between a reference fibre vector and its deformed counterpart complicate the mathematical description of the multiscale mechanics of soft tissues by means of continuum approaches. Such a description would be key for studies on the effect of macroscopic loads on the cell or fibre scales. For example, mechanobiological models involving cell-matrix interactions (e.g. Obbink-Huizer et al. 2014; Loerakker et al. 2016; van Kelle et al. 2019) or those dealing with changes of microstructure due to fibre overstretching, preconditioning and damage (e.g. Balzani et al. 2006; Ehret and Itskov 2009; Sáez et al. 2011). Although these models provide a good phenomenological representation of the macroscale responses induced by changes at the lower length scales, the information that can be inferred about the state of the microscale components (cell or fibres) could be strongly corrupted by the affinity assumption, since in real tissues cell and fibre strains may be considerably different from the ones obtained with an affine behaviour.

### Fibre reorientation is not a sufficient indicator of affinity

Various microscopy techniques were used to study the fibre reorientation in soft tissues upon application of mechanical loads, and the measured mapping between reference and deformed orientations was analysed to verify the existence of affine kinematics. For example, light microscopy of collagen through the thickness of porcine skin in unconfined compression tests indicated non-affine behaviour (Hepworth et al. 2001). Non-affine deformations of fibres were also assumed in human supraspinatus tendon by interpretation of polarised light microscopy (Lake et al. 2011), in bovine annulus fibrosus through confocal microscopy (Huyghe and Jongeneelen 2011), as well as rabbit, porcine and human arterial tissue by means of multiphoton microscopy (Krasny et al. 2018; Cavinato et al. 2020). Conversely, in the detailed study by Lee et al. (2015), various techniques, including multiphoton microscopy, small angle light scattering and small angle X-ray scattering, were used to measure both collagen fibril and fibre orientations in biaxial tests on bovine mitral valve tissue. The analysis showed that the orientations of collagen at both scales, i.e. for fibrils and fibres, are consistent with the assumption of affinity.

While the degree of affinity may not only be length scale- but also tissue-specific, and moreover dependent on the particular architecture and properties of the non-fibrous matrix constituents (Hatami-Marbini and Picu 2009; Zhang et al. 2013), previous results with numerical models of 2D networks (Chandran and Barocas 2005) suggested that measures based only on orientation do not represent a reliable metric to quantify affinity. In line with these findings, our study clearly shows that the orientation distribution of the fibres congruent with an affine motion does not imply affinity itself. This is underpinned by the comparison between the affine and non-affine DNMs, that reveals extremely different stretch distributions (second row in Fig. 13) but almost identical orientation histograms (first row in Fig. 13) between the two cases. In conclusion, these results indicate that an analysis of the full kinematics, i.e. orientations and stretches, would be required to assess affinity, and that fibre reorientation in line with the affine model merely represents a necessary but not sufficient condition for affine fibre deformations.

### Concluding remarks

In this work, we considered two different approaches, discrete and continuum, to describe the mechanics of soft fibrous tissues across the length scales. Based on parametrisations that capture the macroscopic response of a sample tissue to selected deformed states with similar quality, the implemented DNM and CM were used to predict the deformations of what is termed ‘fibres’ in these models.

Clearly, the DNM and CM used in this work represent particular choices and the results will be specific to these selections to some extent. The network architecture, in particular the fibres’ density, orientation, their length distribution and the network’s coordination number, often identified in reconstituted collagen gels below the value of $z\approx4$ used here (Lindström et al. 2010; Jansen et al. 2018), are known to affect the network response (Islam and Picu 2018; Davoodi Kermani et al. 2021). Moreover, non-affine continuum mechanical approaches have been proposed to model fibre network materials (e.g. Raina and Linder 2014) to overcome limitations of the affine approach. Nevertheless, the models studied herein stand for the key characteristics of the DNM and CM approaches, primarily the difference between energy minimisation and kinematic prescription that determine the ‘fibre’ deformation, respectively. Taking for granted that continuum models are by definition limited in describing characteristics at lengths scales below which the continuum hypothesis applies, our study follows a structured and rigorous quantification of the potential errors and misinterpretations occurring when these approaches are applied to model the fibre scale kinematics in soft biological tissues.

Large efforts have been made recently to investigate in dedicated experiments whether the deformation of the collagenous structures in soft biological tissues is affine or non-affine. In this regard, a major result of our analyses points at the importance of considering the full fibre kinematics, i.e. spatial orientations and stretches, for the assessment of affine vs. non-affine motion of fibres. This implies that DIC techniques, mainly resting on the analysis of the change of orientation to date, should also track changes in fibre lengths upon deformation, even if this poses a clear technical challenge.

With regard to modelling, our findings emphasise that continuum models are not only able to capture the macroscopic behaviour, but also provide meaningful insights to fibre reorientation. The interpretation of the stretch, energy or force of what is called a ‘fibre’ in the CM in terms of real fibres in a collagenous network and the use of these metrics as cell-scale stimuli in mechanobiological models, however, remains a risky interpretation across the length scales, unless the affinity, or any other modelling assumption on the fibre motion, has been verified.

## Data Availability

The DNM data related to this article can be found online at the following link https://doi.org/10.3929/ethz-b-000513593.
